# Pitavastatin-loaded bilosomes for oral treatment of hepatocellular carcinoma: a repurposing approach

**DOI:** 10.1080/10717544.2022.2120925

**Published:** 2022-09-08

**Authors:** Maged Kharouba, Amal El-Kamel, Radwa Mehanna, Eman Thabet, Lamia Heikal

**Affiliations:** a Department of Pharmaceutics, Faculty of Pharmacy, Alexandria University, Alexandria, Egypt; b Medical Physiology Department, Faculty of Medicine, Alexandria University, Alexandria, Egypt; c Center of Excellence for Research in Regenerative Medicine and its Applications CERRMA, Faculty of Medicine, Alexandria University, Alexandria, Egypt

**Keywords:** Repurposing, pitavastatin, anti-cancer, liver carcinoma, HepG2, Caco-2, 3D spheroid

## Abstract

Albeit its established efficacy as an anti-hyperlipidemic agent, pitavastatin (PIT) has been shown to have other various therapeutic effects. One of these effects is the anti-cancer activity against hepatocellular carcinoma (HCC). This effect has been evaluated in this study for the first time *via* its oral delivery loaded in bilosomes both *in vitro* in hepatocellular carcinoma (HCC) cell line; HepG2 and *in vivo* in an Ehrlich ascites carcinoma (EAC) model. Moreover, the impact of surface modification of bilosomes with lactoferrin (LF) as an active targeting ligand for HCC was investigated. Bilosomes were prepared by thin-film hydration and different molar phospholipid to bile salt ratios were used to optimize the bilosomal formulation. The molar phospholipid to bile salt ratio was adjusted to 4:1 at pH 7.4. LF-coated bilosomes possessed a particle size, PDI, entrapment efficiency, and zeta potential of 112.28** **nm ± 6.35, 0.229 ± 0.06, 90.56% ± 3.22, and −7.86 mV ± 1.13, respectively. LF-coated bilosomes also increased permeation of PIT when tested on Caco-2 cells by 3.1-folds (compared to uncoated ones or free PIT solution). It also improved the cytotoxicity of HepG2 spheroids 44-folds more than PIT-free solution. RT-PCR analysis showed that LF-coated PIT-loaded bilosomes caused an improvement (2-fold increase) in the apoptotic potential of PIT mediated by caspase-3. In conclusion, the optimized LF-coated PIT-loaded bilosomes were cytotoxic to HCC with improved hepatocytes permeation and cellular uptake. Thus, the proposed formula could be a promising treatment for HCC.

## Introduction

Liver cancer is ranked as the second leading cause of cancer-related death worldwide (Sia et al., 2017). There are different types of liver cancer including intrahepatic cholangiocarcinoma, fibrolamellar carcinoma, hepatocellular carcinoma (HCC), and hepatoblastoma. Among them, HCC is the most occurring, accounting for around 90% of liver cancer cases (Natu et al., 2021). Despite the advances in therapeutic approaches, HCC patients still suffer poor prognosis with relapse rates over 70% (Natu et al., 2021). The risk of HCC varies based on etiology, the extent of liver damage, sex, age, and geographical area (Liver, 2012). HCC is usually associated with risk factors such as chronic viral infections such as type B and C hepatitis viruses, diabetes, obesity, excessive alcohol and tobacco consumption, heritable syndromes, and aflatoxins (Natu et al., 2021). Nowadays, treatment options for HCC include chemotherapy, liver transplantation, immunotherapy, and surgical resection. Although there are many treatment options, none of them can completely cure HCC. Transplantation and resection are considered to be curative options for HCC, but their use is limited by many factors including patient age, stage of HCC, and presence or absence of cirrhosis. However, recurrency rates are high after liver resection (Liu et al., 2021; Natu et al., 2021). In advanced stages, the first-line treatment for HCC according to guidelines is sorafenib with an improvement of only 3 months in the median survival of patients (Abdelmoneem et al., 2019). In most cases, discontinuation of sorafenib occurs due to the devastating side effects that occur during treatment. Therefore, more research should be conducted for more effective and safer alternatives to the present treatment modalities.

Pitavastatin, also known as NK-104, is a lipophilic potent member of the statin family that is widely used for hypercholesterolemia. It is structurally similar to 3-hydroxy 3-methylglutaryl coenzyme A (HMG-CoA); therefore, it binds and inhibits the enzyme utilized in HMG-CoA formation, HMG-CoA reductase enzyme. Inhibition of HMG-CoA reductase enzyme prevents the formation of mevalonate which is essential for cholesterol production, leading to increased internalization of LDL and more expression of LDL-receptor mRNA. The structure of PIT contributes to its unique superior pharmacological effects compared to other statins. Recently, PIT was repurposed for its anti-cancer activity toward various types of cancer including lung (Hu et al., 2020), pancreatic (Chen et al., 2020), ovarian (De Wolf et al., 2018), breast (Bytautaite and Petrikaite, 2020), and liver cancer (You et al., 2016). Its anti-cancer activity in HCC was not fully understood, but research owes its anti-cancer activity to decreased growth of liver cancer cells, caspase-dependent induction of liver cancer cells apoptosis (You et al., 2016), and inhibition of TNF-alpha induced inflammation in HCC (Wang et al., 2006). Additionally, PIT is considered class II drug according to the Biopharmaceutics Classification System (BCS), and suffers from poor solubility and bioavailability (Ashfaq et al., 2022).

Lipid-based vesicular systems are extremely important for the delivery of drugs, as they have a wide range of benefits, including the ability to encapsulate lipophilic and hydrophilic drugs and the ability to overcome many inconveniences such as drug insolubility and drug loading (Li et al., 2019). Bilosomes are advantageous compared to the other lipid-based vesicular system, showing superiority in many aspects. Bilosomes are liposomes incorporating bile salts within their phospholipid bilayer, producing a self-assembling structure. Interestingly, bilosomes have been able to overcome many challenges encountered by the conventional lipid-based vesicular systems such as GIT instability due to the inclusion of the bile salts in the phospholipid bilayer which protect the system from stomach degradation (He et al., 2019; Imam et al., 2022). Moreover, bilosomes also have superior permeation through intestinal membranes when compared to other delivery systems (Saifi et al., 2020).

To further improve the efficacy of the PIT-loaded bilosomes, passive and active targeting have been introduced. Firstly, passive targeting which is highly dependent on the widely known enhanced permeability and retention (EPR) effect. The EPR phenomenon is the guiding principle for better delivery and retention of the anti-cancer nanoparticle to the tumor site to achieve a selective and efficient treatment outcome (Wu, 2021). Although passive targeting could yield successful accumulation of the drug within the tumor, it is limited by numerous factors such as particle size and surface charge. Nanoparticles with a size below 200 nm and carrying a negative surface charge are ideal candidates for successful passive targeting (Liu et al., 2021). On the other hand, active targeting could be achieved by coating the prepared bilosomes with the mammalian hydrophilic cationic protein; LF which belongs to the transferrin family. LF is a natural ligand that can extensively bind to multiple receptors in hepatic cells including asialoglycoprotein receptors (ASGP-R) and lactoferrin receptors (Pireddu et al., 2018). It has been reported that ASGP-R mediated therapy can yield efficacious outcomes in HCC treatment (Zhang et al., 2016). LF was successfully implied to modify the surface of nanocarriers for improving their cellular internalization *via* LF receptor (LFR) endocytosis overexpressed in multiple tumors such as lung, liver, and breast cancer cells (Elzoghby et al., 2020).

It Is worth noting that, this study is the first investigating and evaluating the oral bilosomes encapsulating the repurposed anti-cancer PIT for passive and active targeting of the loaded vesicles to the targeted HCC cells. We utilized the cationic LF that was electrostatically deposited on the surface of anionic bilosomes, owing to its affinity to the mentioned receptors that were overly expressed on HCC cells. The LF layer offered a tumor-targeting potential and added to the stability and efficacy of the formed bilosomes where LF was reported to be stable when administered orally when used for coating of protein nanospheres in treatment of HCC (Abdelmoneem et al., 2018). The efficacy of PIT-loaded bilosomes was explored by both the developed 3D *in vitro* cell culture model (spheroids) altogether with the conventional 2D model; Moreover, *in vivo* animal studies were conducted using Ehrlich ascites carcinoma (EAC) model to evaluate the anti-tumor effects of the selected formulation.

## Materials and methods

Pitavastatin calcium (purity > 98%) was obtained from Baoji Guokang Bio-technology Co, Ltd, China. Lipoid® S100 (l-α-phosphatidylcholine) was gifted from Lipoid AG (Ludwigshafen, Germany). Sodium deoxycholate (SDC), sodium cholate (SC), sodium taurocholate (STC), and Coumarin-6 dye were purchased from Sigma-Aldrich (St. Louis, Mo, USA). Lactoferrin (LF) was kindly donated by Westland Milk products (Hokitika, New Zealand). Dulbecco’s Modified Eagle’s Medium (DMEM) high glucose, heat-inactivated fetal bovine serum (FBS), streptomycin/penicillin, and other cell culture materials were purchased from Lonza Verviers SPRL, Belgium). 3-(4,5-dimethylthiazol-2-yl)-2,5-diphenyltetrazolium bromide (MTT) was purchased from SERVA Electrophoresis GmbH (Germany). TRIzol® Reagent and Hoechst 33342 were purchased from Invitrogen, Thermo Fisher Scientific (Waltham, MA, USA). HPLC grade acetonitrile and chloroform were purchased from Fischer Scientific (Loughborough, UK). Human cancer cell lines (HepG2, Caco-2) were obtained from the Center of Excellence for Research in Regenerative Medicine and its Applications (CERRMA), Faculty of Medicine, Alexandria University, Alexandria, Egypt. PCR primers were purchased from Eurofins Scientific (Luxembourg). One-step RT qPCR Kit (SYBR Green with low ROX) was purchased from Enzynomics Co. Ltd., Yuseong-gu, Daejeon, Korea. All the other reagents were analytical or chromatography grade and used without further purification.

### Quantitative analysis of PIT

UV spectrophotometry and HPLC method were both validated for quantitative analysis of PIT. For UV spectrophotometry, PIT stock solution of 0.1 mg/mL was prepared (10 mg PIT in 100 mL phosphate buffer saline (PBS) of pH 7.4). Serial dilutions were performed to construct a calibration curve at a concentration range of 11–36 μg/mL. The absorbance of the different sample solutions was measured spectrophotometrically (Agilent Technologies-1260 Infinity, Germany) at 240 nm (Vadia et al., 2008). PIT concentration was then determined by fitting the absorbance into the regression equation obtained from the calibration curve. Method Validation was assured by the evaluation of specificity, linearity, and accuracy.

A validated and reported HPLC method for drug assay was also used but with slight modifications (Kumar et al., 2011). The quantitative determination of PIT was performed using an HPLC system (Agilent Technologies-1260 Infinity, Germany), equipped with a UV-variable wavelength detector (G1314F) set at λ_max_ 240 nm, a reversed-phase C_18_ column (Agilent HC-C_18_, 4.6 × 250 mm, 5 μm particle size) and Agilent ChemStation® software 32-bit version (revision B.02.01 SR1). The mobile phase flow rate was 1.0 mL/min at a controlled temperature of 25 °C. The mobile phase consisted of acidified water containing 0.1% (v/v) orthophosphoric acid and acetonitrile at a ratio of 40:60. The sample injection volume was 20 µL. The calibration curve was constructed using peak area vs concentrations in the range of 3-8 μg/mL. Validation parameters such as linearity, accuracy, and precision were also determined.

### Preparation and optimization of PIT-loaded bilosomes

#### Preparation of PIT-loaded and unloaded bilosomes

The bilosomes were prepared by the thin-film hydration method as described by Chen *et al.*, but with slight modifications (Chen et al., 2009). In brief, soybean phosphatidylcholine (SPC, 200 mg) and PIT (10 mg) were co-dissolved in 10 mL of chloroform in a 50 mL round conical flask. The mixture was refluxed at 45 °C to remove the solvent under reduced pressure using a rotary evaporator (Buchi AG, Switzerland). Meanwhile, 10 mL of PBS (pH of 7.4) containing the bile salt (50 mg) were then added to the conical flask for hydration and allowed to mix for 20 min until the milky appearance (hallmark for bilosomes formation) was achieved (Anwekar et al., 2011). Particle size reduction was achieved using both probe sonication (5 min in an ice bath, at 60% frequency, 10 sec on & 2 sec off) using a probe sonicator (Bandelin, berlin, Germany) and bath sonication (5 min at room temperature) using a bath sonicator (ELMA, Germany). The bilosomes were then allowed to stabilize overnight at 4 °C. Same procedure was performed to prepare unloaded vesicular bilosomes where only SPC was dissolved in 10 mL chloroform and the method was continued as previously described

#### Optimization of bilosomes

To optimize bilosomes, two factors have been adjusted; the type of bile salt used and the SPC: bile salt ratio. Bile salts used were sodium deoxycholate (SDC), sodium cholate (SC), and sodium taurocholate (STC). SPC: bile salt ratios assessed were 2:1, 4:1, and 6:1. A 2*3 asymmetrical factorial design was adopted to choose the optimum formulation (B1-B9) where the effect of two independent variables (types of bile salt and SPC: bile salt ratios) and two dependent variables (particle size and PDI) were statistically analyzed using ANOVA.

#### Preparation of LF-coated bilosomes

LF (20–200 mg) was dissolved in 1 mL of PBS (pH 6.5). One mL of bilosomal formulation (2 mg/mL) was added in a dropwise manner to LF solution with continuous gentle magnetic stirring (Thermofisher scientific, Massachusetts, United States) for 2 h. The resultant coated formula was then used for further physicochemical characterization.

### Physicochemical characterization

#### Entrapment efficiency

Entrapment efficiency was performed for LF-coated loaded and uncoated loaded formulae. Measurements were performed in triplicates. Entrapment efficiency was performed using a dialysis tube by 2 methods; Firstly, in the classical dialysis tube method, 1 mL of the formula (1 mg/mL) was added to the dialysis tube (Visking 36/32, 28 mm, MWCO 12,000–14,000; Serva, Heidelberg, Germany), tied from both ends and then soaked into 15 mL of PBS (7.4 pH) to ensure sink condition. The preparation was left in the refrigerator for 2 h (Elmoslemany et al., 2012). The eluent was measured for unentrapped drug using the established spectrophotometric method at 240 nm. Secondly, using the centrifuge method as described by Swarnakar *et al.* (Swarnakar et al., 2014). Simply, 1 mL of the formula (1 mg/mL) was added to the dialysis tube and placed in a falcon tube filled with 15 mL PBS. Centrifugation was performed at room temperature and 500 rpm for 30 min using a high-speed cooling centrifuge (Sigma, Germany). The eluent was measured for unentrapped drug using the same spectrophotometric technique at 240 nm. The entrapped drug was calculated using the following equation Eq. (1):

(1) $$\mathrm{Entrapment}\mathrm{efficiency}\%(EE\%)=(\frac{\mathrm{PIT}\mathrm{total}-\mathrm{PIT}\mathrm{unentrapped}}{\mathrm{PIT}\mathrm{total}})x100$$

Where ‘PIT_unentrapped_’ indicated unentrapped drug concentration and ‘PIT_total_’ indicated total drug concentration in the dispersion.

#### Particle size, PDI, and zeta potential

For the determination of particle size, PDI, and zeta potential, the formula (1 mg/mL) was diluted 50 folds with PBS (7.4 pH) and was subjected to bath sonication for 2 min. Measurements of particle size, PDI, and zeta potential were performed using Malvern zeta sizer Nano ZS (Malvern Instruments, UK) at a wavelength of 633 nm and 25 °C. Measurements were performed in triplicates.

#### In vitro drug release

The *in vitro* release of PIT from LF-coated bilosomes and uncoated bilosomes was performed in comparison to 1 mg/mL free drug solution in PBS (pH 7.4) using the classical dialysis tube method (Nasr et al., 1998). One mL of 1 mg/mL formula or drug solution was filled in a dialysis tube (Visking 36/32, 28 mm, MWCO 12,000–14,000; Serva, Heidelberg, Germany) and tied properly from both ends. The dialysis tube was fully immersed in 15 mL of PBS to maintain sink conditions. The release study was performed in a shaking thermostatically controlled water bath (37 °C ± 0.5 °C − 100 rpm). At predetermined intervals, aliquots of the release medium were withdrawn and filtered through a membrane filter (Nylon Acrodisc®, 0.45 μm, Gelman Sciences Inc., USA). The release medium was replaced with an equal volume of freshly prepared PBS (pH 7.4) to maintain sink conditions. The samples were analyzed using a spectrophotometric method at 240 nm to determine the amount of PIT released. The cumulative percent of drug released was then calculated using Eq. (2).

(2) % Drug released =(PITreleased/PITtotal) x 100

where ‘PIT_released_’ indicated released drug and ‘PIT_total_’ indicated total drug concentration in the dispersion.

#### Transmission electron microscope

A transmission electron microscope was used to assess the morphological features of the optimized bilosomal formulations and to evaluate the shape of the formed system. Before visualization, freshly prepared samples (1 mg/mL) were diluted with filtered deionized water (1:50) and subjected to bath sonication for 5 min at room temperature. A sample drop was placed onto a carbon-coated copper grid and stained using an aqueous solution of 1% uranyl acetate for 30 s then left to dry out. Afterwards, the stained film was visualized using TEM (TEM, JEM-2100F; JEOL, Tokyo, Japan).

### Cell culture

#### Cell culture models for assessing the anti-cancer activity of PIT

A 2D monolayer model of HepG2 cells (HCC cell line) was used to assess the anti-cancer activity of PIT in its different formulations. 3D spheroid cell culture, which is more physiologically relevant to *in vivo* conditions was only used to confirm its cytotoxic effect. Noraml human fibroblast cell line was used to assess the safety of the prepared bilosomal formulations on normal cells.

##### 2D cell culture model

HepG2 cells or human fibroblasts were cultured in high glucose DMEM supplemented with 2 mM L-glutamine, 10% (v/v) FBS, 100 μg/mL streptomycin, and 100 IU/mL penicillin G. Cells were incubated in a humidified CO_2_ incubator (Thermo fisher scientific, Massachusetts, United States) (5% CO_2_ at 37 °C).

##### Spheroid preparation

HepG2 cells grown in 3D spheroids were prepared using a previously published protocol (Liu et al., 2007). Briefly, a cell suspension of 7 × 10^3^ cells/well was carefully seeded in a 96-well U-bottom cell-repellent plate (Corning, cat#4591, USA). The plate was centrifuged to allow aggregation of the cells in the middle of the plate and placed undisturbed in the incubator for 4 days. After 4 days, the spheroids formation was confirmed using inverted field microscopy (Olympus CKX41SF, Japan) and by gentle manipulation with a pipette tip to show the successful aggregation of the spheroids.

##### In vitro cytotoxicity

The cytotoxic effects of PIT solution, LF-coated loaded and uncoated selected bilosomes, and their counter unloaded bilosomes on HepG2 cells were evaluated using MTT assay on both 2D and 3D HepG2 spheroid models (Kijanska et al., 2004). To assess the cytotoxicity on the 2D cell model, HepG2 cells were seeded as a monolayer into 96-well plates (Greiner Bio-One, Germany) at a seeding density of 7 × 10^3^ cells/well and incubated in an atmosphere humidified incubator with 5% CO_2_ at 37 °C for 24 h to allow adherence. After which, media was replaced with an equal volume of fresh medium containing different concentrations (2.5-200 µg/mL) of the free PIT solution and optimized LF-coated and uncoated bilosomal formulations with and without PIT. After incubation for 48 h, MTT solution (5 mg/mL MTT in PBS) was added to each well. The cells were incubated for 4 h in the dark at 37 °C and 5% CO_2_. After incubation, the media with MTT solution was discarded, and a volume of 100 μL of DMSO was added to each well followed by gentle shaking on an orbital shaker (Heidolph Instruments, Schwabach, Germany) for 10 min to dissolve the formazan crystals. The absorbance at λ_max_ 570 nm (A570 nm) was read using an automated microplate reader (BioTek® Instruments, VT, USA). Cell viability was calculated using Equation. (3) and the results were depicted as % cell viability, with 100% representing control untreated cells. All experiments were carried out in triplicates. Consequently, calculation of the 50% of growth inhibitory concentration (IC_50_) was done using a plotted dose-response curve using non-linear regression analysis using GraphPad Prism (version-7.04).

(3) % Cell viability =(Atreated/Acontrol)*100

where, A_treated_ represents the mean absorbance of treated cells and A_control_ represents the mean absorbance of control untreated cells.

A similar MTT experiment was carried out on human fibroblast cells to assess the safety of bilosomes on normal cells. The second generation tetrazolium dye, XTT was used to assess the cytotoxicity on the 3D spheroidal model using a 96-well U-bottom cell-repellent plate (Kiartivich et al., 2017). Spheroids were only used to assess the cytotoxicity of free PIT solution, LF-coated, and uncoated PIT-loaded bilosomes with a concentration range depending on the IC_50_ of each formulation calculated from the 2D cytotoxicity experiment. The XTT solution was dissolved in media (1 mg/mL) on a hot plate (Thermo scientific Cimarec, Massachusetts, United States) and the intermediate electron acceptor, Phenazine methosulfate was added (1.25uL/mL) just before use for complete reduction of XTT. After incubation of the spheroids with the different PIT formulations for 48 h, the XTT solution was added to the wells for 4 h after which the absorbance of the formed color was directly read at 450 nm using a microplate reader.

#### Cellular uptake study

The optimized bilosomal formulations were prepared and loaded with coumarin-6 instead of PIT to assess the cellular uptake of the different formulations on the 2D HepG2 model. HepG2 cells (5 × 10^5^ cells/well) were seeded on a cover slip placed in a 6-well plate. The cells were incubated for 24 h and then treated with either free coumarin-6 dye solution or coumarin-6-loaded in the optimized formulations of LF-coated and its uncoated analogue. The media was aspirated at the end of the incubation time, and the wells were washed twice with PBS and then fixed for 15 min at room temperature with 4% v/v paraformaldehyde solution in PBS. The cells were then rinsed again with PBS, and cellular uptake was observed by confocal laser scanning microscope (CLSM) (Leica® Microsystems Inc. Model DMi8, Metzler, Germany) at the excitation wavelength of 355 nm. To avoid the detrimental effects of ambient light, all procedures were carried out away from direct light. Confocal images were visualized and quantified using ImageJ 1.52a software developed by the National Institutes of Health, USA.

#### Apoptosis assay (Annexin-V-FITC/propidium iodide assay)

Annexin-V-FITC assay on HepG2 2D model) was used to assess the apoptotic effect of PIT in its different formulations (Arya et al., 2018). Samples investigated were free PIT solution, LF-coated bilosomes (unloaded and PIT-loaded), and uncoated bilosomes (unloaded and PIT-loaded). Briefly, cells were incubated at a density of (2 * 10^5^ cell/well) in a 6-well plate (Corning, NY) and left to adhere in CO_2_ incubator for 24 h at a temperature of 37 °C. Hereafter, cells were treated with a dose equivalent to half the IC_50_ calculated from the cytotoxicity experiment; 13.35 µg/mL for uncoated loaded bilosomes, 1.41 µg/mL for LF-coated loaded bilosomes, and 62.3 µg/mL for free PIT solution. Cells were then trypsinized, collected by centrifugation at 2000 rpm, and stained with Annexin V-FITC and propidium iodide according to the manufacturer’s protocol. Analysis of apoptotic cells was performed by 20.000 cells gating by flow cytometer (BD FACS Calibur™ flow cytometer (San Jose, USA)). The experiment was done in triplicates and representative images were provided.

#### *In vitro cellular transport and* permeation study

Caco-2 cells (colorectal adenocarcinoma cell line) were cultured in high-glucose DMEM supplemented with 10% fetal bovine serum (FBS), 100 μg/mL streptomycin, and 100 IU/mL penicillin G and incubated at 37 °C and 5% CO_2_ in a humidified CO_2_ incubator.

##### Cellular transport across Caco-2 monolayer

Caco-2 cells were seeded on a translucent PET filter inserts (ThinCertTM insert, Greiner Bio-One, Germany) having a 113.1 mm^2^ culture surface and a 0.4 μm pore size (apical chamber) at a seeding density of about 10^5^ cells/well. Inserts were added onto the 12-well Transwell® cell-culture plates where 2 mL of the medium was added in the basolateral chamber and 600 μL in the apical chamber. The medium was refreshed every two days for 21 days to ensure complete contact cellular monolayer formation. The integrity of Caco-2 monolayer was confirmed by means of Hoechst 33342 staining assay using a confocal laser scanning microscope. Prior to the transport experiment, the culture medium was withdrawn, Transwell® inserts and receiver chambers were rinsed with Hank’s Balanced Salt Solution HBSS, then pre-incubated in HBSS at 37 °C for 15 min in a CO_2_ incubator. Hereafter, a dose of 62.5 μg/mL of free PIT solution (PBS, pH 7.4), 13.35 μg/mL uncoated loaded bilosome suspension, and 1.4 μg/mL of the LF-coated loaded bilosomes suspension (doses equivalent to half the IC_50_ values of each formulation predetermined in the 2D cytotoxicity experiment) were pipetted into the apical side of the chambers separately. At predetermined different time intervals, 0.25, 0.5, 0.75, 1, 2, 4, and 24 h, an aliquot of 200 μL was withdrawn from the basolateral side of the chambers and replenished with an equal volume of freshly prepared pre-warmed HBSS. Permeated amount of PIT that passed through the basolateral side was analyzed by the HPLC method. Experiments were held in triplicates. The apparent permeability coefficient (P_app_) of PIT solution and the optimized bilosomes was then calculated using equation (4) (Cárdenas et al., 2017).

(4) $$P\text{app}=(\frac{dc}{\text{dt}})*(\frac{1}{A})*C0$$

where:

- P_app_: Apparent permeability coefficient (cm/s)
- dc/dt: Cumulative concentration of drug (c) transported to the basolateral chamber as a function of time (t) and was estimated from the slope of the linear portion of the concentration vs. time plot ▪ A: Surface area of the monolayer filter
- C_0_: Initial concentration of drug in the apical chamber (μg/mL)

### In vivo evaluation of PIT anti-cancer activity

#### Animals

*In vivo* studies were conducted on 56 Swiss albino male mice (7–8 weeks, 25 ± 5 g) housed in stainless steel mesh cages divided in eight groups of 7 mice each, under standard conditions of relative humidity, light illumination, and temperature. They had free access to standard laboratory water and food all over the study. All procedures were strictly performed according to a protocol that was approved by the Animal Care and Use Committee of the Faculty of Pharmacy, Alexandria University, Alexandria Egypt (06-2020-10-12-1-79).

#### Induction of liver tumor

Male Swiss albino mice (7–8 weeks of age, 25 g) were placed in a safe pathogen-free environment. They were provided with normal chow and water. A parent line of Ehrlich ascites carcinoma (EAC) cells was purchased from the National Institute of Cancer, Egypt. They were collected under aseptic techniques from the ascitic fluid of Swiss albino mice harboring 8–10-day old ascitic tumor. Induction of the tumor was conducted as described by Yao *et al*. but with slight modifications (Yao et al., 2003). In brief, a tiny transverse incision was made under the sternum to expose the liver followed by a subcapsular injection of a fixed volume (100 μL) of EAC cells (∼10^6^ of EAC cells) into the right lobe of the liver with a sterile 30-gauge needle until an obvious visualization of transparent blebbing of cells through the liver capsule. Post-injection, a piece of the sterile cotton swab was settled on the site of injection with light pressure for 1 min to avoid bleeding and the escape of EAC cells to the peritoneal fluid to prevent ascites formation. The abdomen was then sutured with a 3/0 nonabsorbable black silk suture. After tumor cell implantation, animals were kept in separate cages and closely monitored for 2 h in a warm cage using cotton padding due to the possibility of post-operational hypothermia. Mice were returned to the animal room after full recovery. Ten days post tumor induction, two mice were randomly selected and terminated to assure solid tumor formation.

#### Evaluation of the anti-cancer activity of PIT

To evaluate the anti-cancer efficacy of PIT-loaded bilosomes, animals were divided randomly into 8 groups (seven mice per group). Negative control (G1, healthy mice injected with saline), positive control (G2, tumor-bearing mice with no treatment), LF-coated PIT-loaded group (G3), LF-coated unloaded group (G4), uncoated PIT-loaded group (G5), uncoated unloaded group (G6), free drug solution in 7.4 pH PBS (G7), IP injected LF-coated PIT-loaded group (G8). All groups received their treatments *via* oral gavage technique, except for G8, where mice were intraperitoneally injected with our optimum formulation to assess if oral administration influenced the anti-cancer efficacy of PIT. Each mouse was given a dose equivalent to 10 mg/Kg/day of PIT equivalent for each formula once daily for 5 consecutive days followed by 2 days off (Chen et al., 2020).

This treatment regimen was repeated for two weeks. At the end of the treatment, mice were subjected to light ether anesthesia and blood samples were collected from the orbital sinus of the mice, and then sacrificed by cervical dislocation. Moreover, blood samples were centrifuged at 3000 rpm at 4^∘^C for 10 min and plasma was stored at −80 °C for further biochemical testing. Furthermore, the excised tumors were then rinsed with cold saline and divided into 3 parts; the first was homogenized with PBS and preserved at −80 °C for determination of alpha-feto protein (AFP) levels using ELISA assay; the second was homogenized with Trizol reagent and stored in −80 °C for RT-PCR determination of caspase-3 gene; finally, the last part was fixed in 10% v/v neutral formaldehyde and embedded in paraffin wax for histological examination.

##### Quantification of different liver biochemical markers

Biochemical markers of HCC were determined using the specified suitable kits; Alanine transaminase (ALT; SGPT) (Biolabo, Cat#80325, France), aspartate transaminase (AST; SGOT) (Biolabo, Cat#80325, France), and serum albumin (Biolabo, Cat#80002, France) along with α-fetoprotein (AFP) (Monobind Inc., Cat#1925-300, USA). All were quantified in different collected plasma samples according to the manufacturer’s protocol.

##### Quantification of tumor growth biomarker using ELISA assay

HCC oncofetal marker; AFP was quantified in liver tumor homogenate using ELISA. Excised liver tumors were homogenized using cold phosphate buffer saline to attain a final concentration of 10% w/v tissue homogenate. AFP was measured using the ‘Mice Alpha-feto protein ELISA Kit’ (Cat#MBS265233, MyBioSource Inc., USA). Quantification was performed according to the manufacturer’s protocol.

##### Quantification of caspase-3 gene expression in liver tissues using RT-PCR

RT-PCR was conducted to evaluate expression levels of mRNA for the caspase-3 gene. Total RNA was isolated from liver tissues homogenized with Trizol reagent (Invitrogen, USA) and purified using the easy spin RNA extraction kit (Intron Biotechnology, India). RNA quality and concentrations were determined using a NanoDrop ND-1000 (NanoDrop DS-11 FX; DeNovix, Delaware, USA). Reverse transcription and real-time quantitative PCR (qPCR)(QuantStudio-1 Real time PCR system, Applied Biosystem, Thermofisher Scientific USA)) were carried out on RNA samples for caspase-3 and GAPDH (as a housekeeping gene) using one-step RT qPCR kit (SYBR Green with low ROX, Intron biotechnology-Korea). The primers used for Caspase-3 and GAPDH genes expression are shown in Table 1. For gene expression quantification, the comparative threshold cycle (ΔΔCt) method was used following Applied Biosystems/Life Technologies’ guidelines. Results were normalized to GAPDH expression and expressed as arbitrary units.

**Table 1.** The primers used for RT-PCR analysis to evaluate gene expression levels of caspase-3 in liver tissues of Swiss albino mice.

Gene	Forward	Reverse
Caspase-3	AGGGGTCATTTATGGGACA	TACACGGGATCTGTTTCTTTG
GAPDH	TCACCACCATGGAGAAGGC	GCTAAGCAGTTGGTGGTGCA

#### Histological examination

Parts of the excised liver tissue from each of the 8 treatment groups were fixed in 10% v/v formaldehyde and embedded in paraffin wax. The specimens were then stained with hematoxylin and eosin staining (H&E) for histological examination using a light microscope (Carl Zeiss, Köln, Germany) – equipped with Canon digital camera.

### Statistical analyses

All experiments were performed in triplicates unless otherwise stated, and the data were expressed as the mean ± standard deviation (SD). Statistical differences were carried out using unpaired Student’s t-test and ANOVA followed by Tukey’s multiple comparison test as post-hoc analysis using GraphPad Prism (Version 7.04, San Diego, CA, USA). Statistical values of *p* ≤ .05 were considered significant.

## Results & discussion

### Quantitative analysis of PIT

The proposed UV spectrophotometric method was successfully applied and validated to quantitatively analyze PIT in bilosomes. The method was considered accurate, specific, precise, and reproducible. Linear correlation was obtained between absorbance and concentration of PIT in the range of 11–36 μg/mL. The linearity of the calibration curve was validated by the coefficient of determination of 0.995. The relative standard deviation (RSD) values of inter-day (1.09–1.38%) and intra-day (0.83–1.12%) variations showed that the used method was reproducible. The LOD and LOQ of PIT were 3.51 and 10.66 μg/mL, respectively. The used method was considered specific, as no interference of the used excipients was detected.

Regarding HPLC assay, the retention time of PIT was 3.6 min. The analytical method was validated concerning linearity, specificity, precision, limits of detection and quantification, and recovery. PIT was quantified from the standard calibration curve, with coefficient of determination (R^2^) of 0.998, and covering linearity concentration range of 3–8 µg/mL. The LOD and LOQ was 2.14 and 6.5 µg/mL, respectively. The intra-day and inter-day precision were less than 1.8%, whereas the % recoveries ranged from 98 to 102%.

### Preparation of bilosomes

Lipid-based vesicular systems are extremely decisive as a drug delivery carrier since they have an immense range of benefits, including the ability to carry both hydrophilic and lipophilic drugs. Recently, bilosomes have been able to overcome plenty of challenges encountered by the conventional lipid-based vesicular systems as the ability to withstand GIT milieu due to the incorporation of the bile salts in the phospholipid bilayer which shelters the system from stomach degradation. Moreover, bilosomes possess better permeation through intestinal membranes than other lipid-based vesicular systems (Ahmad et al., 2017; Saifi et al., 2020). Other advantages include easy preparation techniques, cost-effectiveness, and high stability (Matloub et al., 2018). This study was conducted to encapsulate the lipophilic drug PIT in bilosomes and deliver the system using targeting ligand (LF) to the HCC cells. Bilosomes have been previously studied and proven to be superior to other systems as a drug delivery system to hepatocytes (Pütz et al., 2005). The hepatocyte targeting tendency, improved permeation, GIT stability, and other aforementioned benefits have reinforced the use of bilosomes as a delivery system in our study to target HCC.

#### Optimization of bilosome formulation

Optimization of the prepared blank formulations was performed by evaluating the effect of two factors; the type of bile salt and SPC: bile salt ratio. The type of bile salts investigated were sodium cholate (SC); B1-B3, sodium deoxycholate (SDC); B4-B6, and sodium taurocholate (STC); B7-B9. The SPC:bile salt ratios used were 6:1, 4:1, and 2:1. The zeta potential of all preparations ranged from −12 to −53 mV (Table 2). The negativity of formulations confirmed the deposition of the bile salts within the phospholipid bilayer of the formed bilosomes, imparting the formulation with a negative surface charge (Ahmad et al., 2017; El-Nabarawi et al., 2020)_._

**Table 2.** Composition, particle size, zeta potential, and PDI of blank bilosomes prepared by thin-film hydration method.

Formula	Bile salt	Ratio of SPC:BS	Particle size ± SD (nm)	Zeta potential ± SD (mV)	PDI ± SD
B1	SC	2:1	177.60** **±** **2.91	−12.23** **±** **1.56	0.56** **±** **0.08
B2	SC	4:1	183.26** **±** **11.44	−12.13** **±** **2.30	0.56** **±** **0.08
B3	SC	6:1	196.86** **±** **3.55	−16.86** **±** **2.49	0.68** **±** **0.01
B4	SDC	2:1	120.20** **±** **6.34	−53.70** **±** **1.75	0.41** **±** **0.13
B5	SDC	4:1	83.07** **±** **4.12	−45.32** **±** **1.38	0.15** **±** **0.02
B6	SDC	6:1	95.29** **±** **3.37	−40.40** **±** **1.14	0.42** **±** **0.09
B7	STC	2:1	90.97** **±** **6.05	−29.26** **±** **1.41	0.193** **±** **0.08
B8	STC	4:1	95.09** **±** **8.96	−44.36** **±** **1.22	0.34** **±** **0.04
B9	STC	6:1	132.50** **±** **9.20	−46.96** **±** **3.20	0.53** **±** **0.084

* BS: bile salt, STC: sodium taurocholate, SDC: sodium deoxycholate, SC: sodium cholate, SPC: soybean phosphatidylcholine.

The SC-containing bilosomes (B1-B3) showed an obvious low zeta potential ranging from −12.13 to −16.86 mV. Their particle size and PDI were also statistically higher than other formulations containing SDC (B4-B6) and STC (B7-B9). The zeta potential, particle size, and PDI results came in consistence with those done by Waglewska *et al*. (Waglewska et al., 2020) who have prepared bilosomes using the same preparation method (thin-film hydration) and used SC to stabilize their bilosomes. On the other hand, SDC and STC-stabilized bilosomes showed higher negative values compared to SC bilosomes (El-Nabarawi et al., 2020). Zaki *et al*. have prepared bilosomes containing STC and SDC by thin-film hydration method and hydration was performed using 7.4 pH buffer (Zaki et al., 2021). In accordance with our results, the prepared formulations showed a negative zeta potential of more than −30 mV. The lower zeta potential of SC-containing bilosomes (B1-B3) could be explained by the additional hydroxyl group in the structure of sodium cholate when compared to the structure of sodium deoxycholate. This hydroxyl group could develop steric hindrance that would affect the measured zeta potential (Lee et al., 2005). Moreover, Yang *et al*. have discussed the zeta potential differences among the three bile salts (SC,SDC,STC) and contributed this difference to the bile salt ionization constant (Yang et al., 2011).

The ratio of SPC: bile salt is also a critical factor that can affect the particle size and PDI of formulations (Ahmad et al., 2017). Our results indicated that the ratio of 6:1 (SPC: bile salt) yielded substantially higher particle size than 4:1. The Ratio of 4:1 (SPC: bile salt) can produce bilosomes of significantly lower sizes that were below 200 nm (Chen et al., 2009). In accordance with our results, the formula containing SPC:SDC with a ratio of 4:1 (B5) gave the least particle size, PDI, and zeta potential of 83.07 ± 4.12 nm, 0.15 ± 0.02, and −45.32 ± 1.38 mV, respectively. The highly negative zeta potential could be explained by the phosphatidic acid and the free fatty acids present in the phospholipid (SPC) altogether with the negativity imparted by the bile salt impeded in the phospholipid (Abdelmoneem et al., 2019). Consequently, the formulation prepared using SDC as a bile salt and SPC: SDC ratio of 4:1 (B5) was selected as the optimum formula for further physicochemical evaluation, *in vitro* and *in vivo* studies.

#### Preparation of LF-coated bilosomes

We have developed LF-coated bilosomes encapsulating the repurposed lipophilic PIT to be targeted to HCC cells in the liver. LF is an extensively studied natural cationic ligand that possesses plenty of benefits, including biocompatibility, biodegradability, ease of application as a coating layer owing to its charged nature, and ability to actively deliver the nano system to the targeted liver tumor. LF facilitates the internalization of the bilosomes into cancer cells by both cationic charge-facilitated uptake and its selective binding ability to the various metabolically active receptors that are overexpressed on HCC cells before internalization (Abdelmoneem et al., 2019). The cationic charge-facilitated uptake is based on the cationic nature of LF and the anionic nature of glycosaminoglycans ligands expressed on the surface of different cells (Kanwar et al., 2012). The developed PIT-loaded bilosomes had a particle size and PDI of 83.07 ± 4.12 nm and 0.15 ± 0.02, respectively, and a negative zeta potential of −45.3 mV (Table 3). The coating process of the prepared bilosomes was performed *via* electrostatic interaction between the cationic LF and the anionic bilosomes. The resultant coated bilosomes had a particle size of 112.28 ± 6.35 nm and low negative zeta potential of −7.86 ± 1.13 mV. The increase in the particle size from 83.07 ± 4.12 nm to 112.28 ± 6.35 nm has proved the deposition of the LF on the surface of the bilosomes. The decrease in the negativity of zeta potential was also an indication of a successful LF coating process (Zhang et al., 2019).

**Table 3.** Physicochemical characteristics of coated and uncoated drug-loaded bilosomes in comparison with selected blank formula (using SDC as bile salt and SPC:SPC 4:1).

Formula	Particle size (nm)	PDI	Zeta potential (mV)	EE % (w/w)
Blank bilosome	78.12** **±** **8.92	0.06** **±** **0.01	−42.13** **±** **2.49	–
Uncoated loaded bilosome	83.13** **±** **4.13	0.15** **±** **0.02	−45.32** **±** **1.38	86.51** **±** **4.59
LF-coated bilosome	112.32** **±** **6.31	0.23** **±** **0.06	−7.86** **±** **1.13	90.56** **±** **3.22

**Figure 1.**
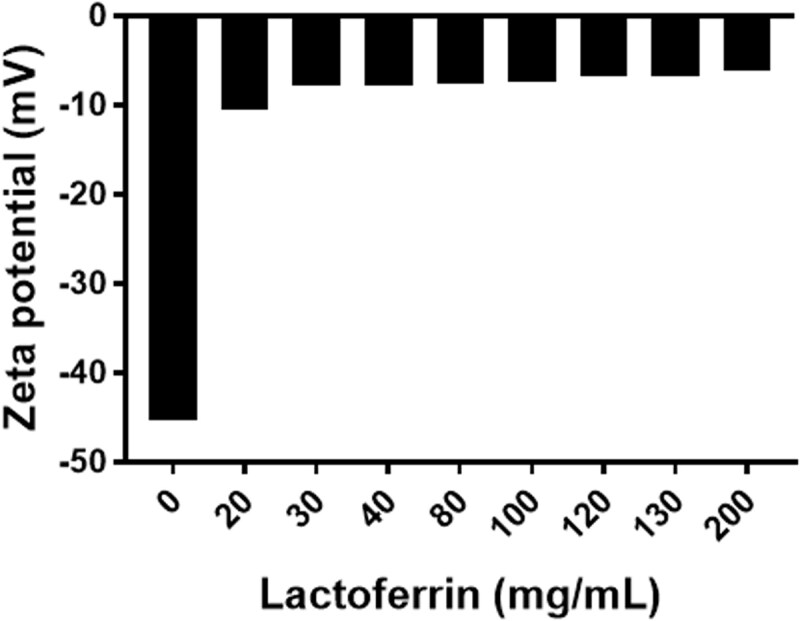
Change in zeta potential upon deposition of the different concentrations of LF (0–200** **mg/mL) on bilosomes for active targeting approach.

The concept of LF coating is based on the electrostatic deposition of the positively charged LF protein and the negatively charged bilosomes (zeta potential of −45.32 ± 1.38 mV). As the concentration of LF increased (20–200 mg/mL), the zeta potential changed toward positivity (Figure 1) reaching a plateau nearly at a concentration of 30 mg/mL. Increasing the concentration of LF above 30 mg/mL did not result in an obvious decrease in zeta potential. Accordingly, LF concentration of 30 mg/mL was used for the coating process.

### Physicochemical characterization

#### Entrapment efficiency

Entrapment efficiency was performed using the classical dialysis tube and was compared to the method developed by Swarnakar *et al.* (Swarnakar et al., 2014). Entrapment efficiency results of both methods yielded similar results taking into consideration that the latter method could provide us with simpler and faster results of entrapment efficiency. There was no significant difference in the entrapment efficiency of the drug in coated and uncoated bilosomes (*p* > .05) where an entrapment efficiency of 86.51% ± 4.59 and 90.56% ± 3.22 was achieved in uncoated and coated formulae respectively.

#### Particle size, PDI, and zeta potential

Particle size is a pivotal attribute of lipid vesicular nanocarriers that can affect system stability, entrapment efficiency, drug release pattern, biodistribution, and cellular uptake (Azhar Shekoufeh Bahari and Hamishehkar, 2016). The particle size of ≤ 150 nm can freely enter or exit the fenestrated capillaries of the tumor microenvironment or to the liver endothelium (Danaei et al., 2018). Particle sizes of the developed formulae (coated and uncoated) were ranging from 83.07 nm ± 4.12 to 112.28 nm ± 6.35. This range of particle size could achieve passive targeting based on enhanced permeability and retention (EPR) altogether with the active targeting achieved by the LF coating. EPR is a phenomenon that enables circulating nanocarriers of a size below 150 nm to extravasate from the blood circulation through the fenestrated tumor capillaries and increase its concentration, and subsequently, the used drug, in the tumor milieu (Li et al., 2016).

PDI is a parameter that is used to show the degree of uniformity of the size distribution of particles. A PDI value of less than 0.3 indicates that the particles have a homogeneous distribution (Danaei et al., 2018). For all prepared formulae (blank, coated, and uncoated), the PDI values were less than 0.22, showing particle homogeneity within the acceptable range.

Zeta potential is a parameter that can be used to predict the physical stability of the formed vesicles (Mohsen et al., 2017). The zeta potential of the different formulae prepared was measured and presented in Table 3. The zeta potential of the blank bilosomes, PIT uncoated bilosomes, and PIT LF-coated formula were −42.13 ± 2.49 mV, −45.32 ± 1.38 mV, and −7.86 ± 1.13 mV, respectively. The high negativity indicated the favorable stability of the formed bilosomes. High zeta potential (> 30 mV) reveals the electrostatic repulsion between similarly charged particles that would hinder particle aggregation, thus improving its physical stability (Abd El-Alim et al., 2019). The slightly higher negativity of the PIT-loaded bilosome could be attributed to the contribution of the negatively charged PIT that is incorporated into the lipid bilayer which increased the surface charge negativity. Due to the electrostatic deposition of the cationic LF layer on the anionic bilosomes, the charge of the LF-coated formula has decreased to-7.87 ± 1.13 mV indicating a successful coating of bilosomes (Abd Elwakil et al., 2018). The slightly negative zeta potential is considered stable as stated by Li et al. (2018) who have prepared an enteric-coated nanoparticle for oral administration carrying a negative charge of −13 mV with good stability. Moreover, the stability of our LF-coated loaded formulation could be attributed to the steric hindrance effect offered by the coating layer. The steric hindrance offered by the LF coatings was reported to be effective in preventing the aggregation of the nanoparticles and subsequently promoting stability of the nano-system (Tian et al., 2022).

#### In vitro release

**Figure 2.**
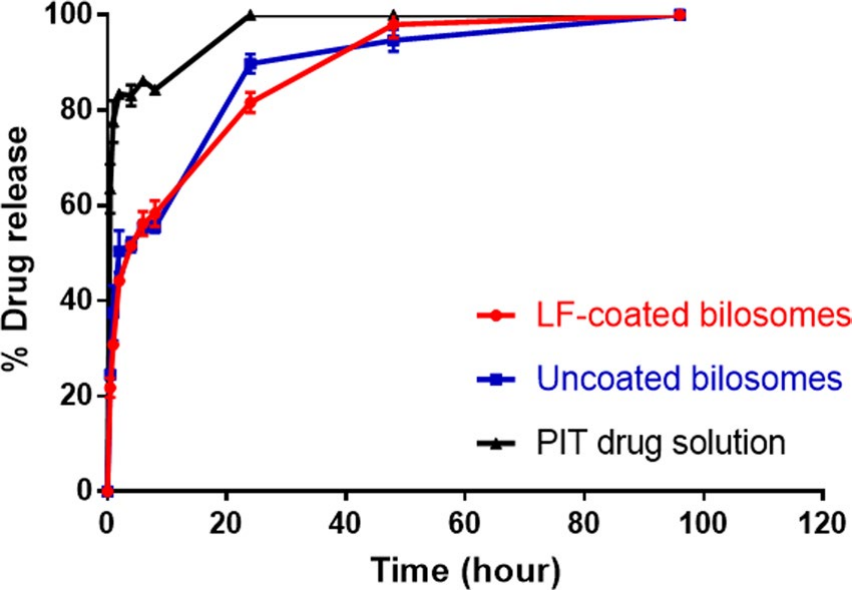
In vitro drug release profile of LF-coated formula, uncoated formula, and free PIT drug solution.

The *in vitro* release profiles of PIT-loaded bilosomes and free drug solution under sink conditions are demonstrated in Figure 2. The release results revealed that the drug efflux from the bilosomes showed a biphasic pattern, having initially burst release followed by a sustained release pattern. It was shown that LF-coated formula released about 21.76 ± 2.04% of the drug during the first 30 min and that was similar to the amount released by the uncoated bilosomes (24.55 ± 1.04%), whereas the free drug solution has released 63.48 ± 5.15% during the same interval. The initial drug burst release of the coated and the uncoated bilosomes could be explained by the presence of the bile salt embedded into the bilosome bilayer that can increase the fluidity of the phospholipid bilayer thus facilitating the lipophilic PIT efflux from the formulation (Kawano et al., 2009). As time proceeded, the release profile for both LF-coated and uncoated showed a release of 81.63 ± 2.11% and 89.77 ± 2.05% drug after 24 h respectively, *p* > .05) in comparison to 100% drug released from drug solution.

#### Transmission electron microscope (TEM)

**Figure 3.**
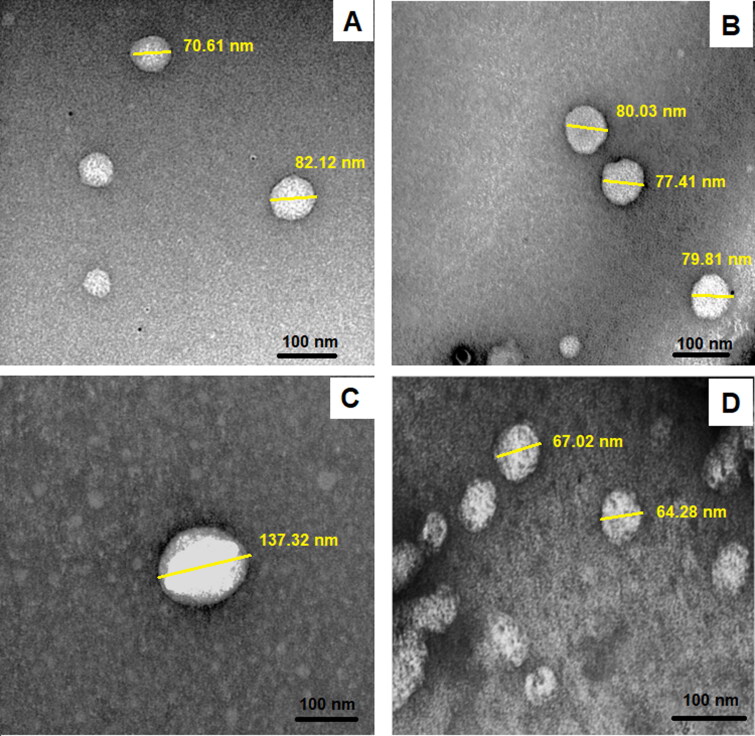
TEM image showing the morphology of uncoated loaded bilosomes (A), uncoated unloaded bilosomes (B), LF-coated loaded bilosomes (C), and LF-coated unloaded bilosomes (D).

Transmission electron microscope (TEM) visualization of the prepared bilosomes (Figure 3) was performed to examine the surface morphology of the vesicular systems. TEM has proven that formulations exhibited a spherical shape. The size of the uncoated bilosomes and LF-coated bilosomes was 70 nm ± 5.36 and 102 nm ± 8.63, respectively. The increase in the size of the coated formulae measured using the TEM proved the deposition of the LF layer on the surface of the bilosomes. The particles also showed no obvious aggregations, which confirmed their stability. The particle size difference between measurements performed by TEM and that of zeta-sizer could be explained by the fact that dynamic light scattering measures the hydrodynamic diameter (nanoparticle and the surrounding liquid layer), whereas TEM measures the actual size of the nanoparticle, yielding a slightly smaller particle size when compared to dynamic light scattering (Kaasalainen et al., 2017).

### Cell culture

#### Cytotoxicity study

**Figure 4.**
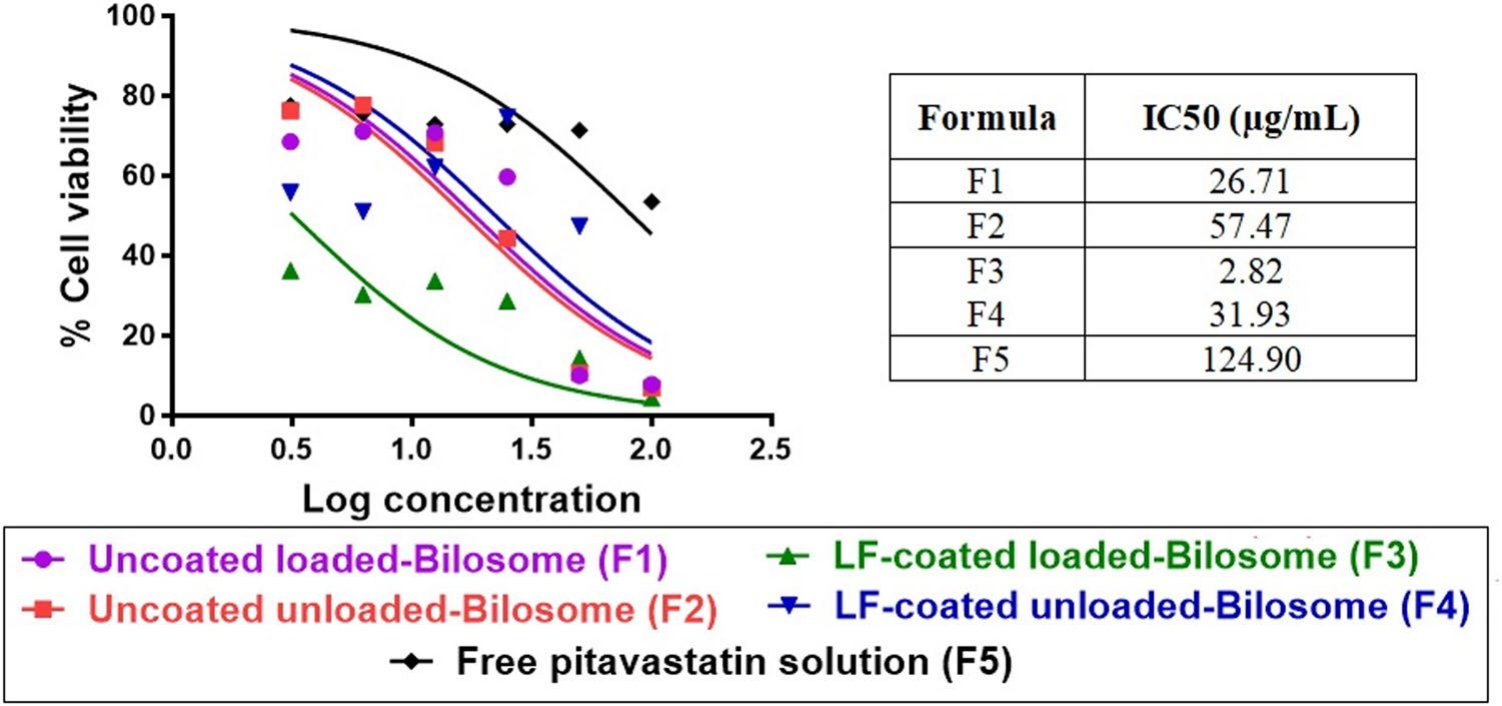
In vitro cytotoxicity studies of various formulations of PIT on 2D HepG2 monolayer cell line using MTT assay. Cells were treated with serial concentrations of PIT-free solutions, LF-coated loaded bilosomes, and uncoated loaded bilosomes and their counter blank formulations equivalent to [2.5–200] µg/mL for 48** **h. Cell viability was measured and IC_50_ for each formula was calculated. Data are expressed as mean ± SD, (n** **=** **3).

The cytotoxic effect of the different prepared formulations was first screened on 2D monolayer HepG2 liver cancerous cells using the MTT test to determine IC_50_ of different formulations (Table 4). By comparing the IC_50_ values (Figure 4), blank uncoated bilosomes showed a more potent cytotoxic effect with a much lower IC_50_ value (57.47 µg/mL) compared to free PIT solution (124.90 µg/mL). This could be attributed to the presence of the bile salt, sodium deoxycholic acid, which is reported to be cytotoxic to HCC cells (Rodrigues et al., 2015). Free PIT showed a very weak cytotoxic effect on HepG2 cells, owing to its high IC_50_ value of 124.90 µg/mL, indicating that PIT alone could not permeate the HepG2 cells and perform its anti-cancer effect. Our results revealed that all prepared bilosomes had superior cytotoxic activity compared to free drug solution. Encapsulating PIT in uncoated bilosomes increased the potency of PIT by 4.7 folds compared to free PIT. On the other hand, LF-coated unloaded bilosome showed higher potency compared to uncoated unloaded bilosomes with a 1.8-fold decrease in the IC_50._ This could be attributed to the inherent anti-cancer effect of LF (Hegazy et al., 2019). LF has been reported to have pharmacological effects on various types of cancer including liver cancer. Moreover, LF-coated loaded bilosomes exhibited the highest cytotoxic effect on the HepG2 cells showing a favorably 44.3, 20.4, 11.3, 9.5-fold increase in potency compared to free drug solution, uncoated unloaded bilosomes, coated unloaded bilosomes, and uncoated loaded bilosomes, respectively. This could be attributed to the presence of both LF and PIT. Herein, LF served a dual purpose: first, it enhanced the anti-cancer effect of the repositioned PIT, as previously described. The second potential role for LF was that it had a preference for certain receptors overexpressed in HCCa cells, such as hepatic lipoprotein receptor-related protein, ASGP-R, and LF receptors (Abdelmoneem et al., 2019). The cytotoxicity assay was also done on human fibroblasts to check the safety on normal cells. The results revealed that the IC_50_ of the used formulas highly exceeded that obtained from the assay when applied on HCC cell line (Table 4). The IC_50_ of F1 (uncoated loaded) on fibroblasts was 140 µg/mL while that of HepG2 cell line was 26.71 µg/mL. Regarding F3 (Coated loaded bilosomes), the IC_50_ was 49.1 µg/mL and 2.82 µg/mL on fibroblasts and HepG2, respectively, knowing that the 2.8 µg/mL concentration when added to the healthy normal cells the viability was about 98%. Also, the F5 (PIT solution) IC_50_ value was 260.8 µg/mL in fibroblasts and 124.9 µg/mL in HepG2 where the latter concentration when applied to fibroblast the viability was about 99%. So, we concluded that the IC_50_ concentrations of the different formulas used to target HCC cells will not have any toxic effect on healthy cells. Accordingly, the results of the cytotoxicity study highlighted the anti-cancer efficacy of the repurposed PIT, the higher permeation and encapsulation ability of the alleged nano-system (bilosomes), and the selective targeting activity of LF.

**Table 4.** Cytotoxicity study comparing IC_50_ results of 2D vs 3D HepG2 cell model and normal human fibroblasts.

	IC_50_ (µg/mL)		
Formula	2D HepG2 cancerous cells	3D HepG2 cancerous Spheroids	2D Normal cells (human fibroblasts)
Uncoated loaded bilosomes (F1)	26.71	61.4	140
LF-coated loaded bilosomes (F3)	2.82	39.8	49.1
Free PIT solution	124.9	169	260

**Figure 5.**
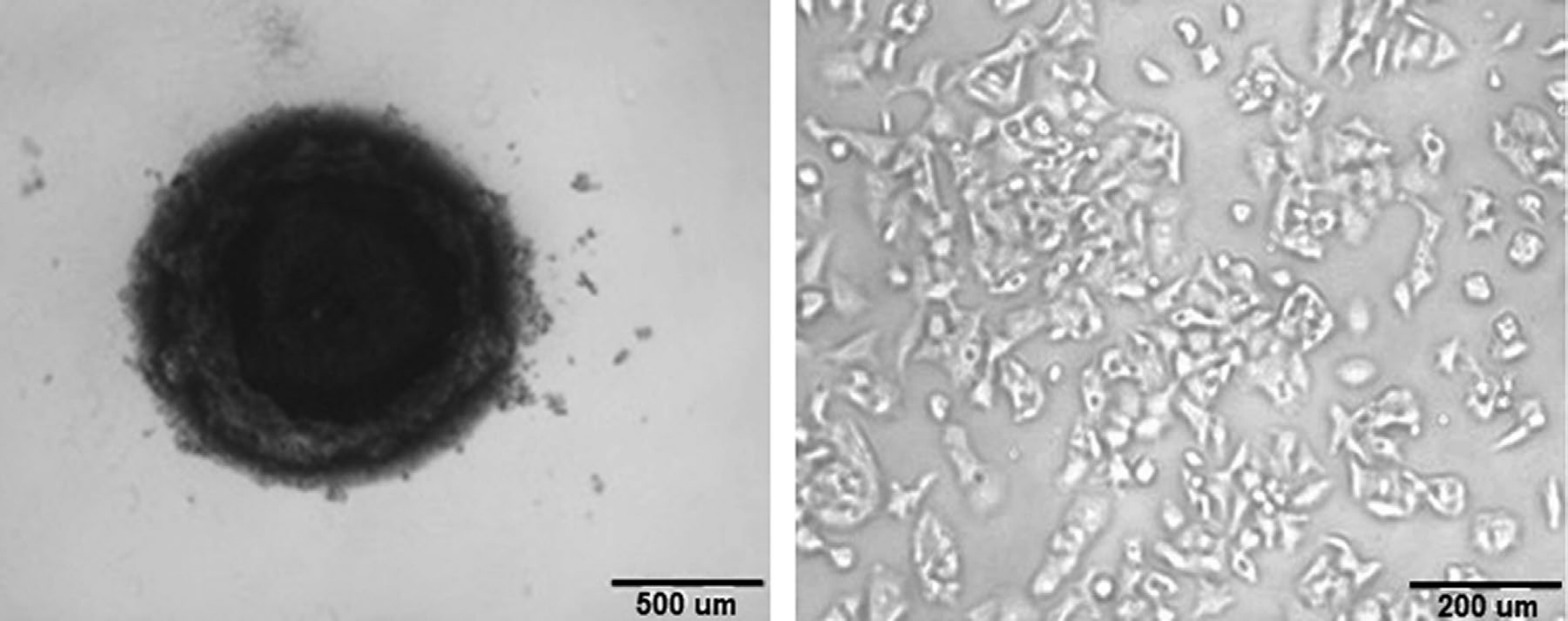
An image representing (a) 3D spheroid cell culture vs (b) 2D HepG2 cells.

The cytotoxic effect of different formulations of PIT was confirmed using 3D HepG2 spheroids. 3D cultures serve as a more relevant model system when compared to 2D models as it better mimics the disease microenvironment helping in the better investigation of the drug’s therapeutic efficacy. In our study, the 3D spheroid of HepG2 cell was successfully prepared and cultured as shown in Figure 5. The spheroids showed a similar cytotoxic pattern when compared to 2D cell culture but with relatively higher IC_50_ values. The highest potency was observed for coated PIT-loaded bilosomes (39.8 µg/mL) followed by uncoated loaded bilosomes (61.4 µg/mL). PIT free solution showed the least potency with IC_50_= 169 µg/mL. The relatively higher IC_50_ values in the spheroids could be attributed to the ability of drug to penetrate 2D cells structure more than 3D cells structure which could also explain why higher doses are required in animal models (Nowacka et al., 2021).

#### Cellular uptake

**Figure 6.**
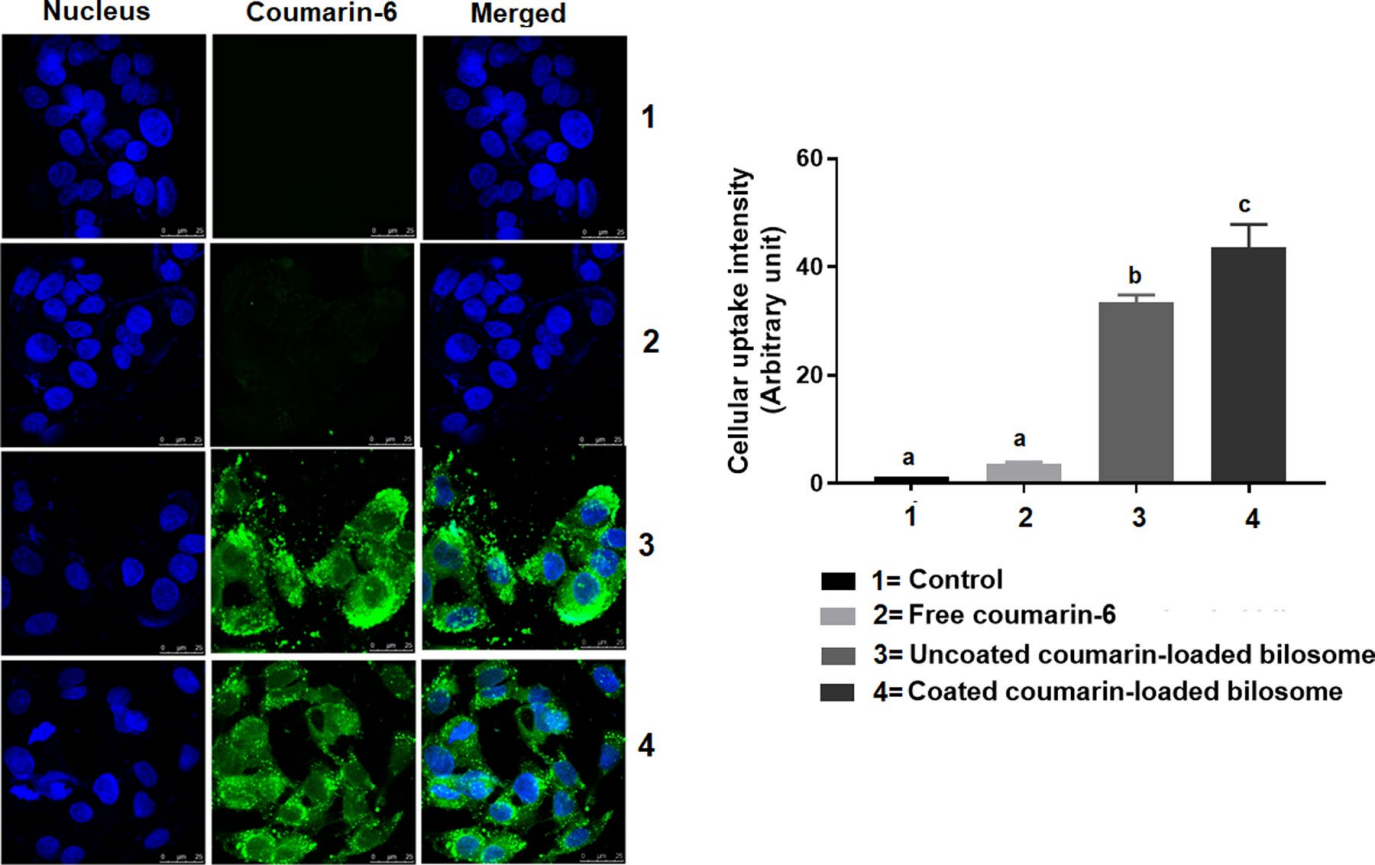
Confocal laser scanning microscope images showing the cellular uptake of three formulations; Free coumarin-6 solution (2), uncoated-loaded bilosomes (3), and LF-Coated loaded bilosomes (4) and compared to the control (1) after 4** **h incubation The images show green fluorescence of coumarin-6 dye and blue fluorescence of nucleus (Hoechst 33342). Statistical significance occurred at level of significance *p*** **≤** **.05 when all groups were compared to the control (1) with mean values a < b < c.

Coumarin-6 was used as a model fluorescent dye to study the effect of different formulations on the drug’s cellular uptake. The cellular uptake of free coumarin solution, LF-coated loaded bilosomes, and uncoated loaded bilosomes to HepG2 cells was based on quantifying the inherent green fluorescence of coumarin-6 dye (Figure 6). The free coumarin solution possessed a minimal uptake behavior, whereas LF-coated loaded bilosomes showed the highest cellular uptake efficacy than its counter uncoated formulation and the free drug solution. The superior uptake of the LF-coated bilosomes could be attributed to the dual activity of both the LF coating which aided in the quicker internalization of the formulation into the HepG2 cells through binding to the overexpressed receptors as mentioned earlier (Abdelmoneem et al., 2019) and to improved cellular permeation of bile salts incorporated in the lipid bilayer of bilosomes as described by Mahajan et al. (Mahajan and Mahajan, 2012). Moreover, nanoparticle-mediated endocytosis could add up to the improved cellular uptake of the bilosomal formulations due to their small particle size (<200 nm) (Niu et al., 2014).

#### Apoptosis study

**Figure 7.**
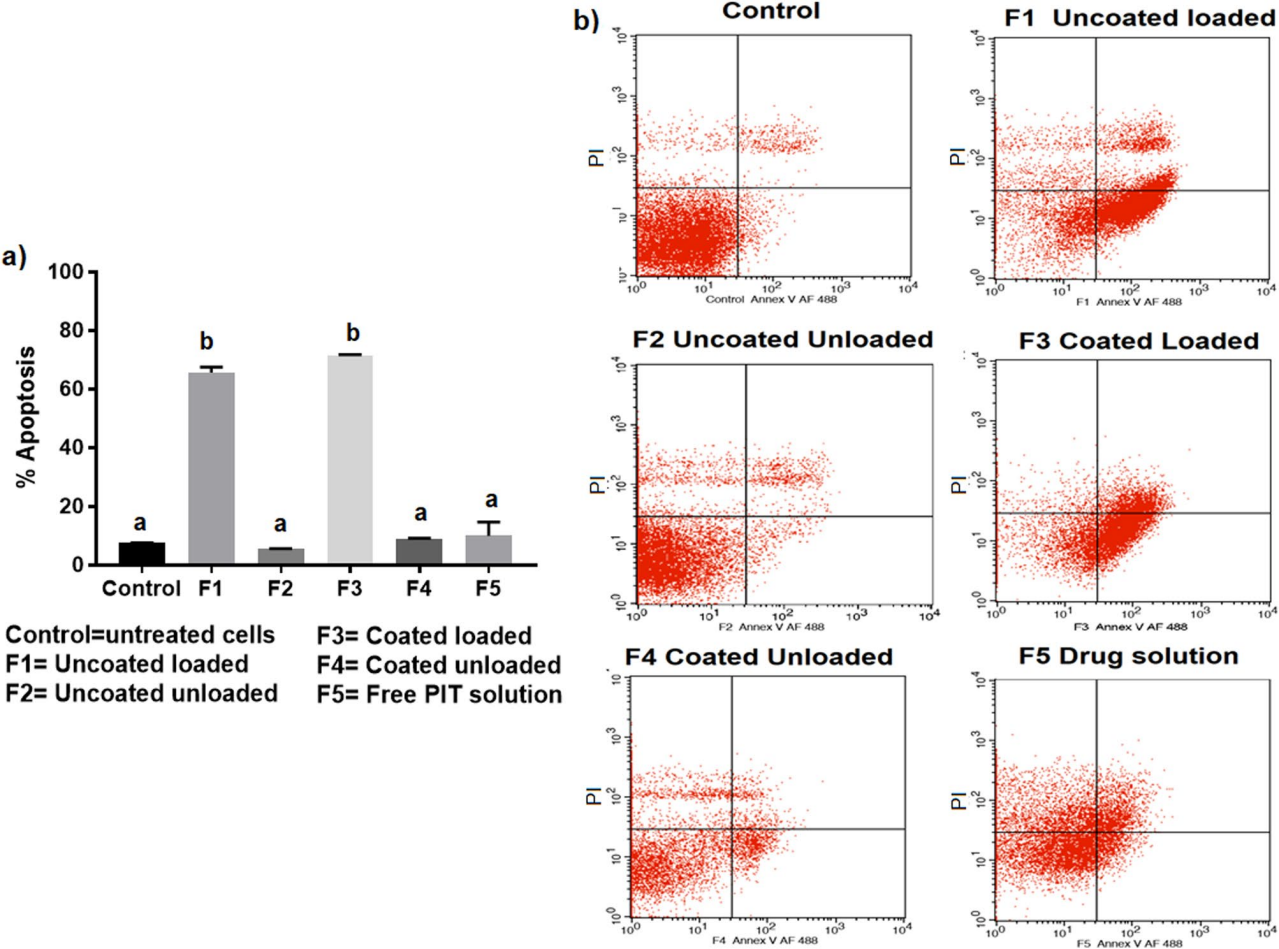
(a) Percentage apoptosis in 2D HepG2 cells after incubation of LF-coated loaded bilosomes(F3) and its counter uncoated bilosomes (F1), LF-coated unloaded bilosomes(F4) and its uncoated analogue (F2), and the free drug solution (F5) for 48** **h. (b) Normal cells served as negative controls. (V (−)/PI (−), living cells; V (+)/PI (−), early apoptosis; V (+)/PI (+), late apoptosis; V (−)/PI (+), necrotic cells). Statistical significance occurred at level of significance *p*** **≤** **.05 when all groups were compared to the control with mean values a < b.

Apoptosis; programed cell death, is an extensively studied phenomenon that has widely been recognized as a major effective and highly selective anti-tumor therapeutic response (Wong, 2011). The therapeutic efficacy of the repurposed PIT against HCC has been recently proposed for its anti-proliferative and apoptotic effects (Wang and Xu, 2007; You et al., 2016). Wang et al. (2006), were the first to highlight the efficacy of PIT in HCC, referring its anti-cancer activity to its inhibitory effect on tumor necrosis factor-alpha (TNF-α) that in return downregulated IL-6 that normally leads to inflammation-associated cancer (Wang et al., 2006). Wang *et al.* (2007) then proposed that the apoptotic effect of PIT is caspase 3-dependent, proving that 10** **µM PIT could cause apoptosis in 50% of HepG2 cells (Wang and Xu, 2007). Ishida *et al*. then proposed that statins, as inhibitors of the mevalonate pathway, possess anti-cancer properties through apoptosis by inhibiting the formation of geranylgeranyl pyrophosphate (GGPP) and farnesyl pyrophosphate (FPP); products of the mevalonate pathway that are required in cell membrane localization of proteins. Accordingly, their downregulation leads to apoptotic activity (Ishida et al., 2016). Recently, You *et al*. supported the previously postulated anti-cancer hypothesis of PIT where he proved that the major anti-tumor effect of PIT was *via* its anti-proliferative and apoptotic ability (You et al., 2016). In our study, we explored the effect of PIT encapsulation in bilosomal formulations on improving its cellular apoptotic effect. Remarkably, blank unloaded bilosomal formulations (F2 and F4) and free PIT solution (F5) showed insignificant difference (*p*** **>** **.05) in apoptotic activity when compared to the control group as evident in Figure 7, indicating that cell apoptosis was only induced *via* enhancing the anti-cancer activity of PIT through bilosomal encapsulation. Coated loaded (F3) and uncoated loaded (F1) formulations showed the highest apoptotic activity with 71.53** **±** **0.24% and 65.63** **±** **1.64% apoptosis, respectively, compared to only13.71** **±** **8.92% for free PIT solution (F5). Consequently, these results verified that bilosomes significantly induced higher cellular apoptosis compared to free PIT solution, which ultimately aided in augmenting the anti-cancer effect of PIT for management of HCC.

#### Cellular transport and permeation through caco-2 cells

**Figure 8.**
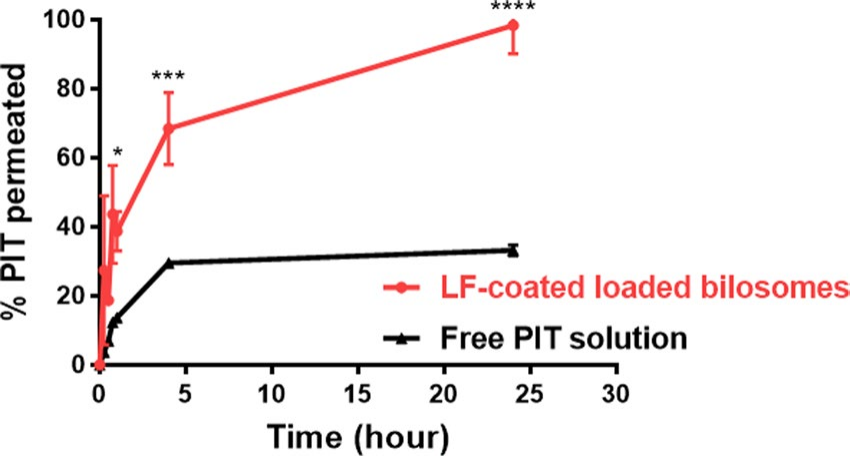
Permeation studies of PIT-LF-coated loaded bilosomes and free drug solution across Caco-2 cell monolayer. Data are expressed as mean ± SD, (n** **=** **3). LF-coated formula showed statistically significant results where ****p** **≤** **.0001 when compared to free PIT solution.

The Caco-2 cell cellular transport model, which simulates the *in vivo* permeation through the small intestine epithelium, is considered a crucial tool for inspecting the drug permeation rate (Matsson et al., 2005). Hence, the utilization of the Caco-2 model for the *in vitro* evaluation of the permeability of drug-encapsulated bilosomes is considered an asset to infer their oral bioavailability. PIT-free solution and LF-coated bilosomes were individually dispersed in the upper compartment and the cumulative amounts of PIT permeated through Caco-2 monolayers were calculated at different time intervals over 24** **h to depict their transport across the caco-2 cells toward the lower compartment. As seen in Figure 8, LF-coated bilosomes displayed a significantly higher (*P*** **≤** **0.0001) permeation rate of 98.3% ±6.73 of PIT that was transported across the Caco-2 monolayer after 24** **h. Conversely, free drug solution showed lower PIT transport rates of 33.21% ±1.25 after 24** **h. This finding could be attributed to the pronounced competence of bilosomes to open intercellular tight junctions and pass through the paracellular pathway which was supported by the opening of cellular tight junction proteins including Zonula occludens-1 (ZO-1), F-actin as previously reported (Niu et al., 2014). Concerning the better permeability of bilosomes, Ahmed *et al.* have postulated that bilosomes make tight junctions leakier by sequestering the calcium ions responsible for maintaining the integrity of the tight junctions (Ahmed et al., 2020), whereas Niu *et al.* have proved that bilosomes permeate Caco-2 cells through paracellular transport mechanism owing to the reversible opening of tight junctions of Caco-2 cells as observed by CLSM. It was visualized as the redistribution of filaments of actin and the discontinuous appearance of ZO-1 proteins, indicating the opening of the tight junctions and subsequently the paracellular transport of bilosomes (Niu et al., 2014). Moreover, McClean *et al.* also postulated that formulations with particle size below 120** **nm exhibit transcellular and paracellular transport routes, and that applied to our selected formulae particle size (McClean et al., 1998). Accordingly, these results proved the prominent potential of bilosomes to enhance the permeability of PIT when orally administered compared to a free drug solution.

### Assessment of anti-cancer efficacy of different PIT formulations in vivo

**Figure 9.**
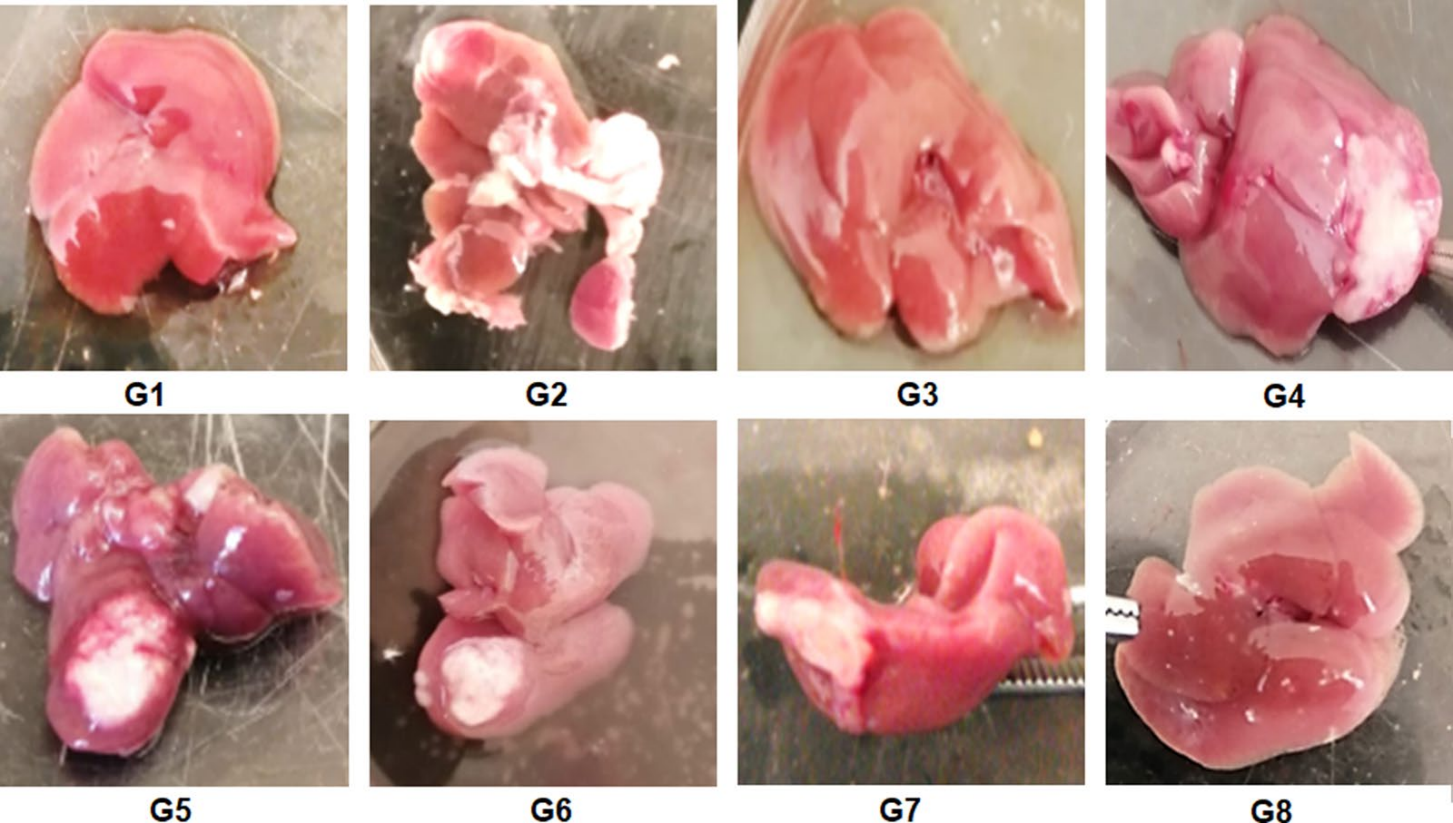
Liver of the terminated mice of the 8 study groups; G1 (negative control), G2 (positive untreated control), G3-7 treated groups with oral administration of different formulae where G3 (LF-coated loaded), G4 (LF-coated unloaded), G5 (uncoated unloaded), G6 (uncoated unloaded), G7 (free PIT solution) and G8 (Same formula as in G3 ‘LF-coated loaded formula’ but administered IP rather than orally).

This study aimed to elucidate the anti-cancer efficacy of the different bilosomal formulations encapsulating the repurposed drug PIT in an EAC model. The injection of EAC cells in the right lobe of the liver has developed a solid palpable tumor where its size has been monitored by weighing the whole liver in randomly selected mice from each group terminated 10** **days post-injection. For the first time in this study, the effectiveness of the LF-coated PIT-loaded bilosomes was assessed after both oral and IP administration to check if the GIT has affected the efficacy of the bilosomal formulation. As shown in Figure 9, at the end of treatment, the liver of mice treated with free PIT solution (G7) and blank coated and uncoated formulations (G4, G6, respectively) together with the positive control group (G2) showed an obvious increase in tumor size (about 3 to 4-folds compared to the negative control group (G1). G5 (uncoated loaded formulation) showed 2-fold decrease in the tumor size when compared to the positive control group. On the other hand, G3 and G8 (representing groups treated with orally and IP- administered LF-coated-PIT loaded bilosomal formulations, respectively) showed the least tumor size that was 3-folds less when compared to the positive control group. That could be attributed to the cytotoxic effect of the repurposed PIT along with LF coating strategy to target the liver tumor as previously discussed (Abdelmoneem et al., 2019). There was an insignificant difference (*P*** **>** **0.05) between the tumor burden in both G3 and G8 indicating that oral administration had no negative influence on the effectiveness of the bilosomal preparations.

**Figure 10.**
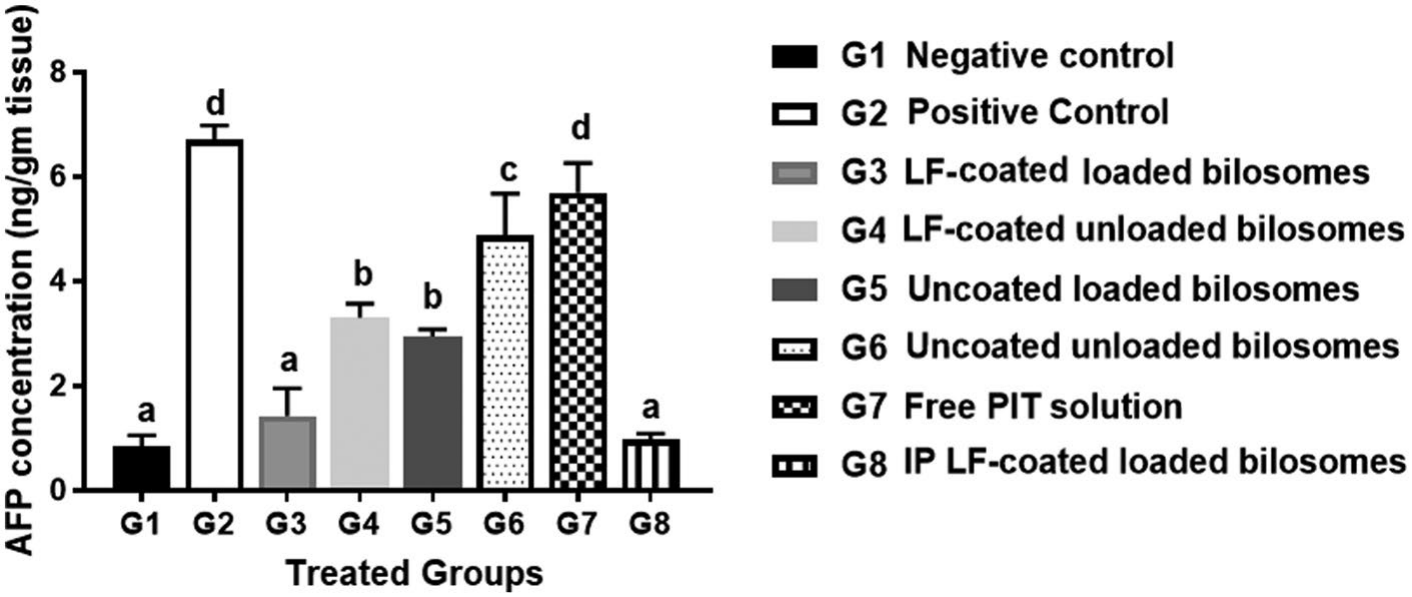
Levels of Alpha-feto protein quantified by ELISA assay of the 8 study groups; G1 (negative control), G2 (positive untreated control), G3-7 treated groups with oral administration of different formulae where G3 (LF-coated loaded), G4 (LF-coated unloaded), G5 (uncoated unloaded), G6 (uncoated unloaded), G7 (free PIT solution) and G8 (Same formula as in G3 ‘LF-coated loaded formula’ but administered IP rather than orally). All treated groups (except G3 and G8) had significantly higher AFP levels compared to the negative group (G1) where a significant difference was achieved at *p*** **≤** **.05 with mean values a** **<** **b < c** **<** **d.

For further confirmation of the anti-cancer efficiency of PIT in different treatment groups, the level of alpha-feto protein (AFP) in excised liver tissue from different treatment groups was quantified using the ELISA assay (Figure 10). AFP is considered the most widely used serum marker for diagnostic and prognostic features of HCC (Ridder et al., 2022). It has been reported that the level of AFP could be correlated directly with tumor growth in mice (Yao et al., 2003). The high AFP levels together with other diagnostic features such as abdominal ultrasound or a history of hepatitis B virus support the HCC diagnosis (Hernandez-Meza et al., 2021). The treatment groups with favorable treatment outcome showed the least levels of AFP (restored to normal levels) where G3 (oral LF-coated PIT loaded formula) and G8 (IP LF-coated PIT loaded formula), showing statistically insignificant (*p*** **>** **.05) difference compared to the negative control (G1), thus further proving cytotoxic predominance of our developed LF-coated formula loaded with PIT on the cancer cells when given either orally or IP. The potential of the different formulae to restore AFP levels was in the following order: G8 > G3 > G5 > G4 > G6 > G7. G4 (LF-coated unloaded) had higher ability to restore AFP levels when compared to G6 (uncoated unloaded formulations), that could be due to the inherent anti-cancer effect of LF (Jiang et al., 2014). Furthermore, free PIT solution (G7) showed statistically insignificant (*p*** **>** **.05) change in AFP results compared to the positive control (G2), owing to their high lipophilicity and poor penetration through the tumor. Consequently, a comparison between loaded formulations and their counter blank (i.e. without drug) bilosomes suggested that PIT possessed an anti-cancer effect which was proven by the lower AFP value for loaded formulae (G3 and G5) compared to (G4 and G6).

#### Biochemical assessment of liver function

EAC model made obvious modifications in the levels of biochemical markers of liver function as serum ALT, AST, AFP, and serum albumin. High ALT and AST levels can reflect hepatic damage and toxicity related to tumor growth (Limdi and Hyde, 2003), whereas albumin levels decline tremendously in presence of liver cirrhosis and cancer (Kawaguchi et al., 2021). It was reported that HCC cause statistically significant elevated levels of AFP, AST, and ALT, yet downgraded levels of albumin (Zaghloul et al., 2017).

**Figure 11.**
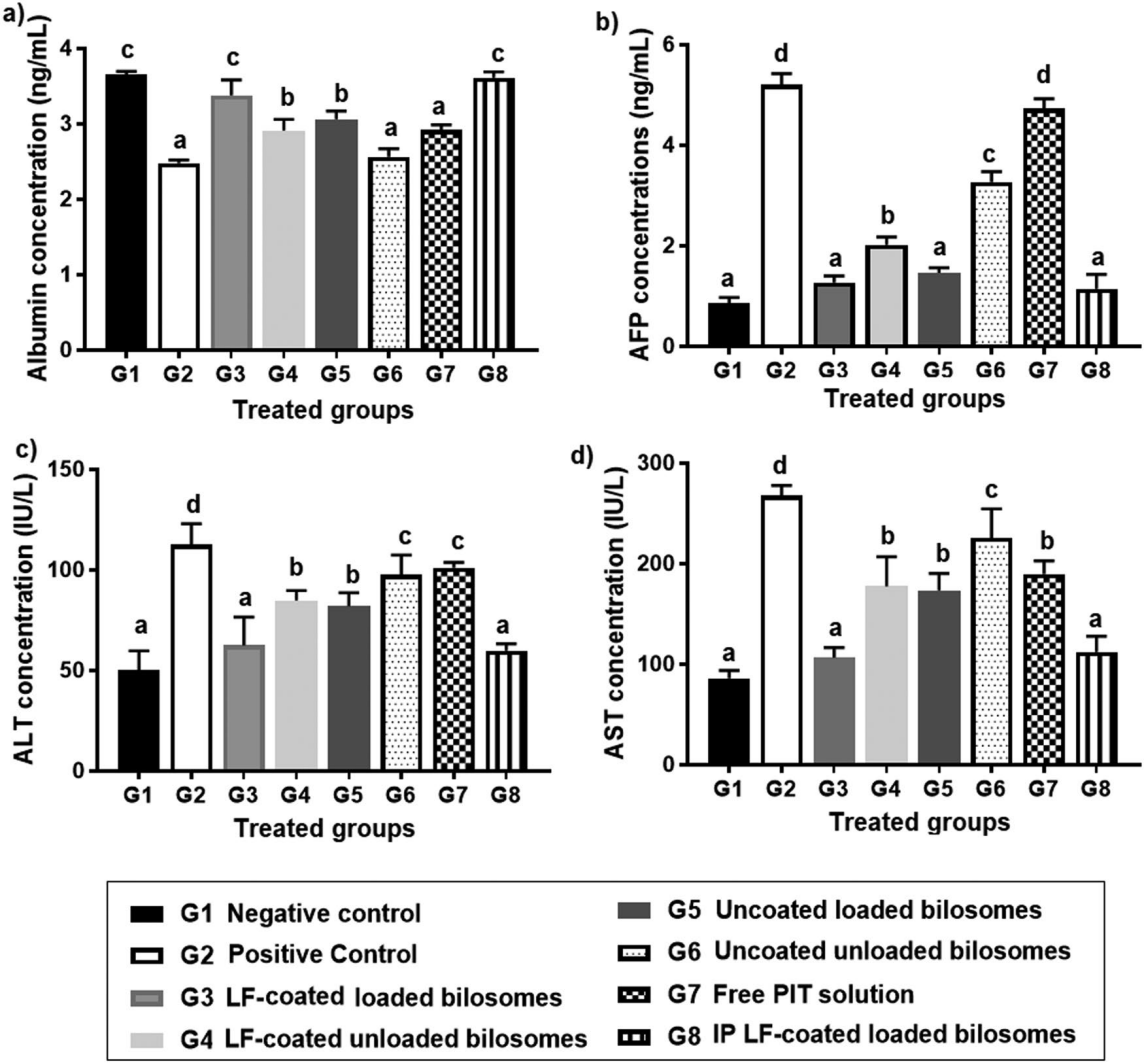
Levels of liver function biochemical markers in the 8 treatment groups; G1 (negative control), G2 (positive untreated control), G3-7 treated groups with oral administration of different formulae where G3 (LF-coated loaded), G4 (LF-coated unloaded), G5 (uncoated unloaded), G6 (uncoated unloaded), G7 (free PIT solution) and G8 (Same formula as in G3 ‘LF-coated loaded formula’ but administered IP rather than orally) showing levels of (a) albumin, (b) Alpha feto protein (AFP), (c) alanine transaminase (ALT) and (d) aspartate transaminase (AST). All results are statistically compared to normal control (G1) where a significant difference was achieved at *p*** **≤** **.05 with mean values a** **<** **b < c** **<** **d.

By comparing the results of positive control (G2) with their counter negative control (G1), we have observed that AFP, ALT, and AST levels have increased by 8.4-, 2.3-, and 3.1-folds, respectively, while albumin levels decreased by 1.5-fold, suggesting tumor formation in G2 (Al-Shahari et al., 2021). The ability to normalize the levels of AFP, AST, ALT, and albumin could help categorize the efficacy of the given formulations in each of the 6 treatment groups (Figure 11). By comparing the results, it was found to be consistent with previous results of AFP levels in cancerous liver tissue as shown in Figure 10 where G3 and G8 showed statistically insignificant difference (*P*** **>** **0.05) when compared to negative control groups. This was attributed to the improved cellular uptake of PIT enclosed in bilosomes and the significant localization of the formed system in the target HCC. Comparing G3 (LF-coated loaded formula) to G4 (LF-coated unloaded) has proved the cytotoxic effect of PIT as indicated by levels of the three measured biomarkers. Results showed a significant increase of 1.4, 1.4, and 1.7-fold for AFP, ALT, and AST, respectively, and a decrease of 1.1-fold for albumin when comparing coated loaded formula (G3) with coated unloaded (G4). The difference between the two formulae was less than expected but that was concluded as the fundamental anti-cancer effect of LF. G3 (LF-coated loaded) caused a decrease by 1.14, 1.31, 2.18-fold for AFP, ALT, AST, respectively, and a 1.1-fold increase in albumin levels, proving that targeting could aid in the overall efficacy of the formulation by facilitating localization of the developed formula in hepatoma cells. Free drug solution (G7) and uncoated unloaded bilosomes (G6), were statistically insignificant (*p*** **>** **.05) when compared to the positive control with regard to albumin and ALT serum levels. For G7, this could be due to the limited uptake of the unencapsulated PIT into hepatoma cells and the absence of the LF targeted approach, while for G6, it might be explained by the absence of PIT altogether with the untargeted approach of bilosomes. Overall results have proven the high potency of the LF-coated loaded formulae (G3 and G8) in treating tumor-bearing hepatoma cells and improved targeting ability.

#### RT-PCR analysis of apoptotic markers

**Figure 12.**
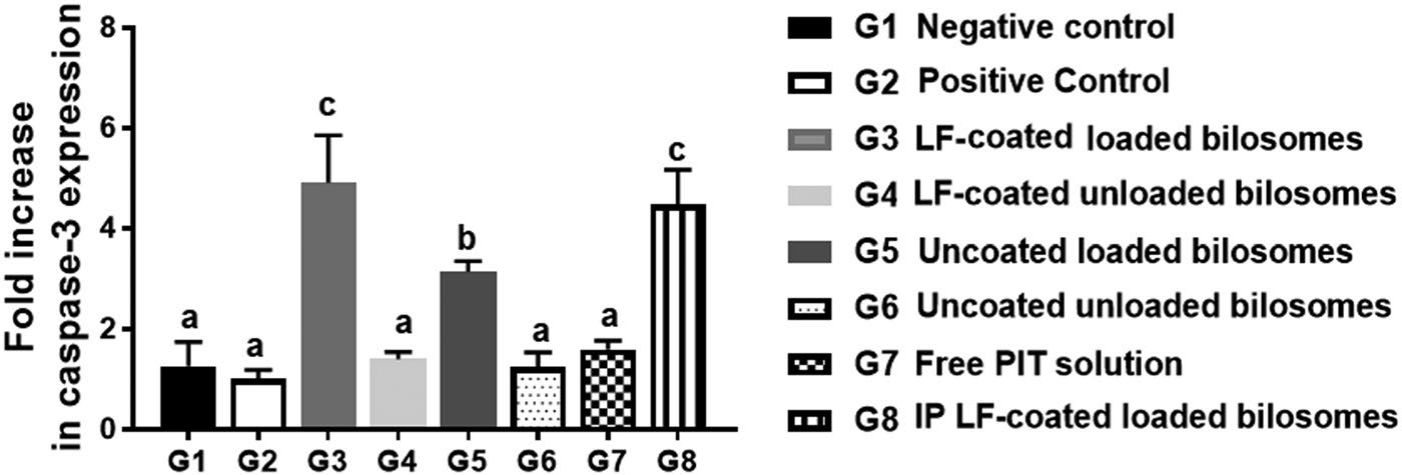
Fold increase in caspase-3 gene expression levels quantified by RT-PCR for the 8 treatment groups; G1 (negative control), G2 (positive untreated control), G3-7 treated groups with oral administration of different formulae where G3 (LF-coated loaded), G4 (LF-coated unloaded), G5 (uncoated unloaded), G6 (uncoated unloaded), G7 (free PIT solution) and G8 (Same formula as in G3 ‘LF-coated loaded formula’ but administered IP rather than orally), normalized to the expression of the housekeeping gene, GAPDH using (2^−ΔΔCt^) method (n** **=** **3). All groups were statistically compared to a negative control (G1) when *p*** **≤** **.05 with mean values a** **<** **b < c.

RT-PCR analysis of the Caspase-3 gene was performed to measure the apoptotic capability of the different treatment groups and thus further proving the cytotoxicity of PIT as an anti-cancer drug on the gene level (Figure 12). It was previously reported that PIT can induce apoptosis in cancer cells *via* the caspase pathway as reported by You et al. (2016). Activation of caspase-3 from procaspase-3 announces the initiation of the apoptosis process in the cancerous cell. Measuring the fold increase of the caspase-3 gene could be informative about the potency of the formula used, where elevated values of caspase-3 highlight a highly potent formula. It was noteworthy that mice groups administered the unloaded formulae (G4, G6) and free drug solution (G7) were statistically insignificant (*p*** **>** **.05) when compared to G1& G2, assuring that none of them can elicit the apoptotic process. PIT solution (G7) low expression of caspase-3 gene (limited apoptosis) might be attributed to the limited cellular uptake of free PIT. G3 and G8 showed 4 to 5-fold increase in caspase-3 levels than the corresponding unloaded formulae (G4), proving the inherent apoptotic potential of PIT that is encapsulated in bilosomes. Bilosomes were beneficial in improving the permeation and uptake of PIT altogether with the targeting power of LF. The inherent anti-cancer effect of LF was not evident in the caspase-3 PCR study, since LF cytotoxic effect was not mediated by caspase-dependent pathway. That was proven by the low fold change in the caspase-3 gene of G4 (coated unloaded) which was statistically insignificant (*p*** **>** **.05) when compared to its uncoated analogue G6 (uncoated unloaded) thus further proving that LF is incapable of initiating apoptotic process. Consequently, PCR of caspase-3 gene proved that G3 and G8 are cytotoxic to HCC cells by inducing caspase-3 apoptotic pathway.

#### Histological examination

**Figure 13.**
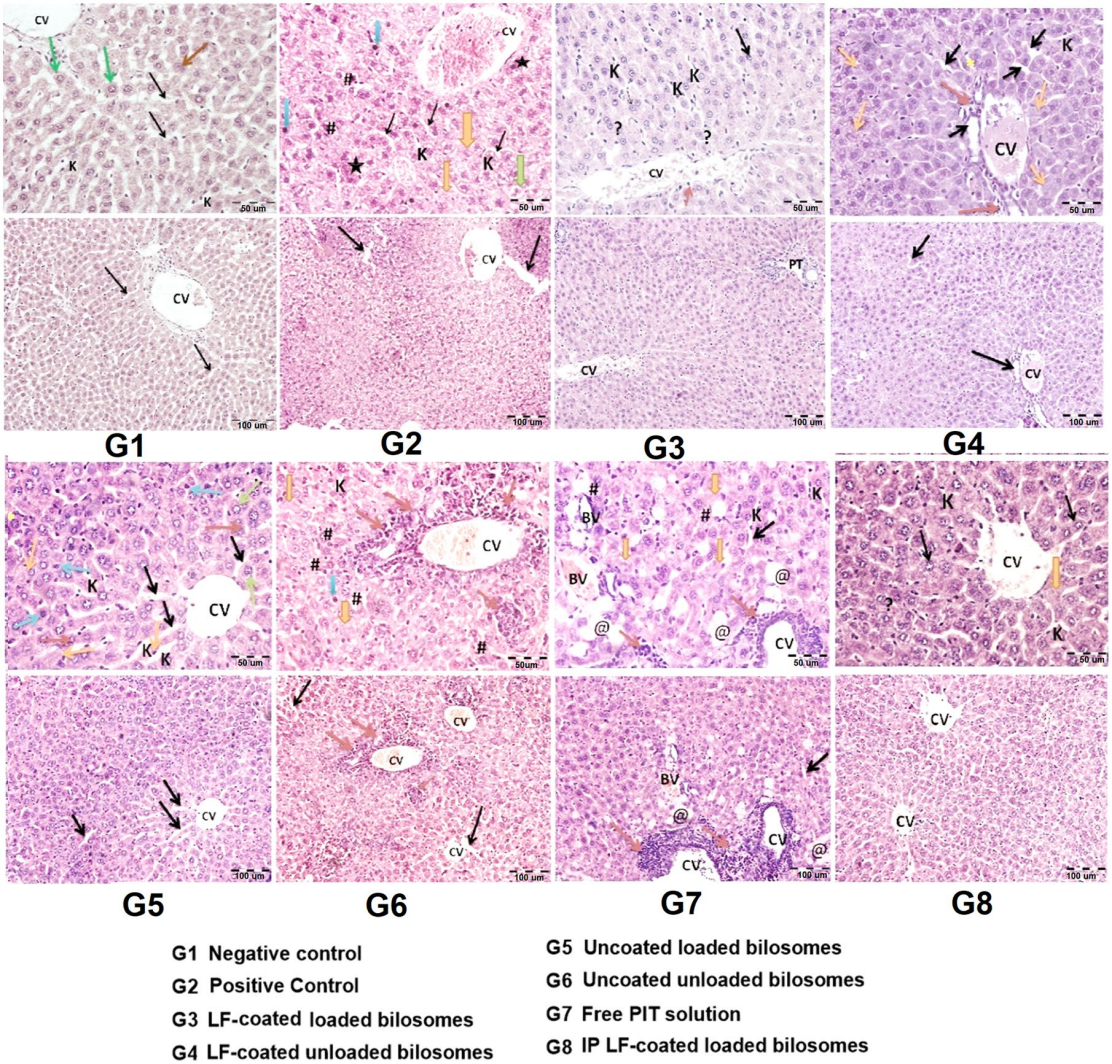
Hematoxylin and eosin staining of liver cancer tissues of 8 study groups; G1 (negative control), G2 (positive untreated control), G3-7 treated groups with oral administration of different formulae where G3 (LF-coated loaded), G4 (LF-coated unloaded), G5 (uncoated unloaded), G6 (uncoated unloaded), G7 (free PIT solution) and G8 (Same formula as in G3 ‘LF-coated loaded formula’ but administered IP rather than orally). (*) hyper eosinophilia, (?) binucleated, (K) Kupfer cells, (#) high N/C ratio, (CV) central vein, (PT) portal vein, (BV) blood vessel. (H&E stain, Mic. Mag. × 200, and × 400).

Sections from normal control groups (G1) showed typical histological features of normal liver cells as shown in Figure 13. Hepatocytes were arranged in cords 1–2 cells thick around the central vein (CV) and separated by blood sinusoids (black arrows). Higher magnification showed Kupffer cells (K) within the sinusoids (black arrows). Hepatocytes were also observed with their classical histological appearance of polyhedral cells with granular eosinophilic cytoplasm (green arrows). Most of hepatocytes were seen with central vesicular nuclei (green arrows) while some were binucleated (brown arrows).

Liver sections isolated from untreated positive control groups (G2) showed disorganized hepatic architecture with thickened hepatic plates and widened hepatic sinusoids (black arrows). The central vein (CV) was congested with the absence of portal tracts. Larger magnification showed hepatocytes with nuclear irregularities in the form of pyknotic/apoptotic nuclei (blue arrows) and karyolysis (orange arrows) as described by El-Wahab *et al.* (El-Wahab et al., 2009). Other cells showed enlarged nuclei (high N/C ratio) (#). Some cells showed hyper-eosinophilia (black star) as well as anaplastic tumor cells with cellular and nuclear variation in shape and size (green arrows). Kupffer cells (K) were less frequently seen within the widened sinusoids (black arrows) (Kapoor et al., 2014). Hepatocytes showed nuclear irregularity. The same features were seen in liver samples for uncoated unloaded groups (G6), coated unloaded groups (G4), and in free PIT solution (G7). Moreover, in PIT solution group (G7), some blood vessels (BV) were seen invading in between the hepatocytes. Some cells showed changes in the form of polygonal cells with ballooned clear cytoplasm and peripheral flattened nuclei (@). On the other hand, treating the mice with uncoated loaded formula (G5) lead to restored general liver architecture where the hepatocytes were arranged in cords 1-2 cells thick around the central vein (CV) and separated by widened blood sinusoids (black arrows) (Alshaymaa et al., 2012), contributing to the anti-cancer effect of PIT. Higher magnification showed the presence of Kupffer cells (k) within the widened sinusoids (black arrows). Hepatocytes were observed with their classical histological appearance of polyhedral cells with granular eosinophilic cytoplasm (green arrows). Most of the hepatocytes were seen with central vesicular nuclei (green arrows) while some were binucleated (brown arrows). Some hepatocytes showed nuclear irregularity in the form of pyknotic/apoptotic nuclei (blue arrows) and karyolysis (orange arrows). Some cells also showed hyper-eosinophilia (black star). Mice treated with LF-coated loaded bilosomal formulations (G3) showed also restored general liver architecture and the hepatocytes arranged in cords 1–2 cells thick around the central vein (CV) and separated by blood sinusoids. This group (G3) showed a superior effect where the portal tracts (PT) appeared well defined at the angles of the classical hepatic lobules. Higher magnification showed restored liver morphology in addition to minimal vascular congestion and very mild mononuclear cellular infiltration (brown arrow) are noticed. These results could be attributed to the role of LF targeting, the anti-cancer effect of PIT, and improved permeation and uptake of bilosomes. IP administration of the LF-coated loaded formulation (G8) showed a similar effect to the orally administered ones (G3), where the hepatocytes were observed with their classical histological appearance of polyhedral cells with granular eosinophilic cytoplasm (black arrows). Most of hepatocytes were seen with central vesicular nuclei (black arrows) while some are binucleated (?). However, few cells still showed karyolitic nuclei (orange arrow).

Based on the above-mentioned results, the superior anti-cancer effect of the prepared bilosomes in our study could be attributed to many factors. Firstly, encapsulation of PIT into bilosomes enhanced its anti-cancer effect, which can cause a reduction of their doses and, consequently, diminish its systemic toxicity. Moreover, the integration of bile salts in the phospholipid bilayer enhanced its resistance to stomach digestion (He et al., 2019), allowing its safe oral administration thus improving patient compliance. Additionally, the small size of our prepared bilosomes enhanced their localization in the tumor tissue *via* the enhanced permeation and retention (EPR) effect. Furthermore, the active targeting capability achieved by LF coating enabled the internalization of bilosomes into hepatoma cells by LF and ASGP receptor-mediated endocytosis together with the LF cationic charge-based internalization. Lastly, the repurposing concept has offered plentiful benefits such as economic benefits, reported safety profiles, and a time-saving approach.

## Conclusion

In this study, for the first time, LF-coated bilosomes were fabricated for efficient delivery of the lipophilic drug; PIT for HCC treatment. All prepared bilosomes demonstrated a desirable small size with high encapsulation efficiency together with improved tissue accumulation due to enhanced cellular internalization *via* active targeting potential of LF. Furthermore, bilosomes enhanced the transport across Caco-2 cells and facilitated cellular uptake. Besides, the LF-coated formula showed a highly favorable potency against HepG2 cells compared to free drug solution and other prepared bilosomes shown in results of both 2d and 3d cell culture models. This obvious superiority was manifested through elevated AFP protein levels together with normalization of hepatic enzymes (ALT & AST), and serum albumin in the Ehrlich ascites carcinoma model. We can conclude from the overall results of both *in vitro* and *in vivo* studies, that the PIT-encapsulated LF-coated bilosomes offered a promising potent anti-tumor formula against HCC over free PIT that can be administered orally without compromising drug efficacy.
